# Metrics for evaluating 3D medical image segmentation: analysis, selection, and tool

**DOI:** 10.1186/s12880-015-0068-x

**Published:** 2015-08-12

**Authors:** Abdel Aziz Taha, Allan Hanbury

**Affiliations:** TU Wien, Institute of Software Technology and Interactive Systems, Favoritenstrasse 9-11, Vienna, A-1040 Austria

**Keywords:** Evaluation metrics, Evaluation tool, Medical volume segmentation, Metric selection

## Abstract

**Background:**

Medical Image segmentation is an important image processing step. Comparing images to evaluate the quality of segmentation is an essential part of measuring progress in this research area. Some of the challenges in evaluating medical segmentation are: metric selection, the use in the literature of multiple definitions for certain metrics, inefficiency of the metric calculation implementations leading to difficulties with large volumes, and lack of support for fuzzy segmentation by existing metrics.

**Result:**

First we present an overview of 20 evaluation metrics selected based on a comprehensive literature review. For fuzzy segmentation, which shows the level of membership of each voxel to multiple classes, fuzzy definitions of all metrics are provided. We present a discussion about metric properties to provide a guide for selecting evaluation metrics. Finally, we propose an efficient evaluation tool implementing the 20 selected metrics. The tool is optimized to perform efficiently in terms of speed and required memory, also if the image size is extremely large as in the case of whole body MRI or CT volume segmentation. An implementation of this tool is available as an open source project.

**Conclusion:**

We propose an efficient evaluation tool for 3D medical image segmentation using 20 evaluation metrics and provide guidelines for selecting a subset of these metrics that is suitable for the data and the segmentation task.

## Background

Medical 3D image segmentation is an important image processing step in medical image analysis. Segmentation methods with high precision (including high reproducibility) and low bias are a main goal in surgical planning because they directly impact the results, e.g. the detection and monitoring of tumor progress [1–3]. Warfield et al. [4] denoted the clinical importance of better characterization of white matter changes in the brain tissue and showed that particular change patterns in the white matter are associated with some brain diseases. Accurately recognizing the change patterns is of great value for early diagnosis and efficient monitoring of diseases. Therefore, assessing the accuracy and the quality of segmentation algorithms is of great importance.

Medical 3D images are defined on a 3D grid that can have different sizes depending on the body parts imaged and the resolution. The grid size is given as (*w*×*h*×*d*) denoting the width, height, and depth of the 3D image. Each 3D point on the grid is called a voxel. Given an anatomic feature, a binary segmentation can be seen as a partition that classifies the voxels of an image according to whether they are part or not of this feature. Examples of anatomic features are white matter, gray matter, lesions of the brain, body organs and tumors. Segmentation evaluation is the task of comparing two segmentations by measuring the distance or similarity between them, where one is the segmentation to be evaluated and the other is the corresponding ground truth segmentation.

Medical segmentations are often fuzzy meaning that voxels have a grade of membership in [0,1]. This is e.g. the case when the underlying segmentation is the result of averaging different segmentations of the same structure annotated by different annotators. Here, segmentations can be thought of as probabilities of voxels belonging to particular classes. One way of evaluating fuzzy segmentations is to threshold the probabilities at a particular value to get binary representations that can be evaluated as crisp segmentations. However, thresholding is just a workaround that provides a coarse estimation and is not always satisfactory. Furthermore, there is still the challenge of selecting the threshold because the evaluation results depend on the selection. This is the motivation for providing metrics that are capable of comparing fuzzy segmentations without loss of information. Note that there is another common interpretation of fuzzy segmentation as partial volume, where the voxel value represents the voxel fraction that belongs to the class. The fuzzy metric definitions provided in this paper can be applied for this interpretation as well.

There are different quality aspects in 3D medical image segmentation according to which types of segmentation errors can be defined. Metrics are expected to indicate some or all of these errors, depending on the data and the segmentation task. Based on four basic types of errors (added regions, added background, inside holes and border holes), Shi et al. [5] described four types of image segmentation errors, namely the quantity (number of segmented objects), the area of the segmented objects, the contour (degree of boundary match), and the content (existence of inside holes and boundary holes in the segmented region). Fenster et al. [6] categorized the requirements of medical segmentation evaluation into accuracy (the degree to which the segmentation results agree with the ground truth segmentation), the precision as a measure of repeatability, and the efficiency which is mostly related with time. Under the first category (accuracy), they mentioned two quality aspects, namely the delineation of the boundary (contour) and the size (volume of the segmented object). The alignment, which denotes the general position of the segmented object, is another quality aspect, which could be of more importance than the size and the contour when the segmented objects are very small.

Metric sensitivities are another challenge in defining metrics. Sensitivity to particular properties could prevent the discovery of particular errors or lead to over- or underestimating them. Metrics can be sensitive to outliers (additional small segmented objects outside the main object), class imbalance (size of the segmented object relative to the background), number of segmented objects, etc. Another type of sensitivity is the inability to correctly deal with agreement caused by chance. This is related to the baseline value of the metric, which should ideally be zero when the segmentation is done at random, indicating no similarity [7].

There is a need for a standard evaluation tool for medical image segmentation which standardizes not only the metrics to be used, but also the definition of each metric. To illustrate this importance, Section “Multiple definition of metrics in the literature” shows examples of metrics with more than one definition in the literature leading to different values, but each of them is used under the same name. In the text retrieval domain, the TREC_EVAL tool^1^ provides a standardization of evaluation that avoids such confusion and misinterpretation and provides a standard reference to compare text retrieval algorithms. The medical imaging domain lacks such a widely applied instrument.

Gerig et al. [8] proposed a tool (Valmet) for evaluation of medical volume segmentation. In this tool only five metrics are implemented. There are important metrics, like information theoretical metrics as well as some statistical metrics like Mahalanobis distance, and metrics with chance correction like Kappa and adjusted Rand index, that are not implemented in the Valmet evaluation tool. Furthermore, this tool doesn’t provide support for fuzzy segmentation. The ITK Library^2^ provides a software layer that supports medical imaging tasks including segmentation and registration. The ITK Library provides evaluation metrics that are mostly based on distance transform filters [9]. However, this implementation has the following shortcomings: First, the ITK Library doesn’t implement all relevant metrics needed for evaluating medical segmentation. Second, since most of the metrics are based on distance transform filters, they are sensitive to increasing volume grid size in terms of speed as well as memory used. One way to reduce this effect is to use the bounding cube (scene) [6, 10], i.e. the smallest cube including both segments, that is to exclude from calculation all background voxels not in the bounding cube. However, there are some shortcomings using the bounding cube, first the bounding cube remains large when the segments are large, far from each other or there are outliers far from the segments; second, the bounding cube affects the results of metrics that depend on the true negatives. In Section “Testing the efficiency”, we show that the ITK implementation of relevant metrics fails to compare segmentations larger than a particular grid size. Since very large medical segmentations, like those of whole body volumes, are already common, this is a significant restriction.

This paper makes the following contributions:

- It provides an overview of 20 evaluation metrics for volume segmentation, selected based on a literature review. Cases where inconsistent definitions of the metrics have been used in the literature are identified, and unified definitions are suggested.
- It provides efficient metric calculation algorithms that work optimally with large 3D image segmentations by taking advantage of their nature as dense distributions of voxels. Efficiency is becoming ever more important due the increasing size of segmentations, such as segmentation of whole body volumes.
- The paper provides fuzzy definitions for all selected metrics. This allows uncertainty in medical image segmentation to be taken into account in the evaluation.
- It provides metrics generalized for segmentation with multiple labels
- It provides an efficient open source implementation of all 20 metrics that outperforms state-of-the art tools in common cases of medical image segmentation

The remainder of this paper is organized as follows: Section “Ethics approval” provides the ethics approval. Section “Evaluation metrics for 3D image segmentation” presents a short literature review of metrics. In Section “Metric definitions and Algorithms”, we present the definition for each identified metric in the literature review as well as the algorithms used to efficiently calculate the metric value. We provide in Section “Multiple definition of metrics in the literature” examples of multiple definition of metrics in the literature that leads to confusion and motivates a standard evaluation tool. Section “Implementation” provides details on the tool implementation, i.e. architecture, programming environment, usage as well as the optimization techniques have been used. Experiments performed to test the tool efficiency are presented in Section “Testing the efficiency”. A discussion about metric properties, bias, and utilities as well as guidelines for metric selection is presented in Section “Metric selection”. We conclude the paper in Section “Conclusion” and give information about the availability and requirements in Section “Availability and requirements”.

### Ethics approval

The images used for this study are brain tumor MRI images and segmentations provided by the BRATS2012 benchmark organized in conjunction with the MICCAI 2012 conference, and whole body MRI/CT scans, provided by the VISCERAL project (www.visceral.eu) that were acquired in the years 2004–2008, where data sets of children (<18 years) were not included due to the recommendation of the local ethical committee number S-465/2012, approval date February 21st, 2013.

### Evaluation metrics for 3D image segmentation

We present a set of metrics for validating 3D image segmentation that were selected based on a literature review of papers in which 3D medical image segmentations are evaluated. Only metrics with at least two references of use are considered. An overview of these metrics is available in Table 1. Depending on the relations between the metrics, their nature and their definition, we group them into six categories, namely overlap based, volume based, pair-counting based, information theoretic based, probabilistic based, and spatial distance based. The aim of this grouping is to first ease discussing the metrics in this paper and second to enable a reasonable selection when a subset of metrics is to be used, i.e. selecting metrics from different groups to avoid biased results.

**Table 1:** Overview of the metrics implemented in this tool

Metric	Symb.	Reference of use in medical images	cat.	Definition
Dice (=F1-Measure)	*DICE*	[1, 2, 15, 16, 57–63]	1	(6)
Jaccard index	*JAC*	[15, 16, 21–23, 59, 60, 62]	1	(7)
True positive rate (Sensitivity, Recall)	*TPR*	[10, 16, 60, 62–64]	1	(10)
True negative rate (Specificity)	*TNR*	[10, 16, 60, 62]	1	(11)
False positive rate (=1-Specificity, Fallout)	*FPR*	→ Specificity	1	(12)
False negative rate (=1-Sensitivity)	*FNR*	→ Sensitivity	1	(13)
F-Measure (F1-Measure=Dice)	*FMS*	→ Dice	1	(15), (16)
Global Consistency Error	*GCE*	[21–23, 65, 66]	1	(17) to (19)
Volumetric Similarity	*VS*	[15, 21–23, 59, 61, 67]	2	(21)
Rand Index	*RI*	[21, 22, 65, 66]	3	(30)
Adjusted Rand Index	ARI	[68, 69]	3	(32)
Mutual Information	*MI*	[2, 32, 57]	4	(33) to (38)
Variation of Information	*VOI*	[21, 22, 65, 66]	4	(39), (35)
Interclass correlation	*ICC*	[8, 70]	5	(41)
Probabilistic Distance	*PBD*	[8, 59]	5	(43)
Cohens kappa	KAP	[1, 62]	5	(44) to (46)
Area under ROC curve	*AUC*	[2, 64, 69]	5	(47)
Hausdorff distance	*HD*	[8, 15, 59, 61–63, 71, 72]	6	(48), (49)
Average distance	*AVD*	[62, 63]	6	(50), (51)
Mahalanobis Distance	*MHD*	[15, 73]	6	(52) to (54)

The symbols in the second column are used to denote the metrics throughout the paper. The column “reference of use” shows papers where the corresponding metric has been used in the evaluation of medical volume segmentation. The column “category” assigns each metric to one of the following categories: (1) Overlap based, (2) Volume based, (3) Pair counting based, (4) Information theoretic based, (5) Probabilistic based, and (6) Spatial distance based. The column “definition” shows the equation numbers where the metric is defined

### Metric definitions and Algorithms

We present the definitions of all metrics that have been implemented. Let a medical volume be represented by the point set *X*={*x*_1_,…,*x*_*n*_} with |*X*|=*w*×*h*×*d*=*n* where *w*, *h* and *d* are the width, height and depth of the grid on which the volume is defined. Let the ground truth segmentation be represented by the partition $S_{g}=\{{S_{g}^{1}},{S_{g}^{2}}\}$ of *X* with the assignment function ${f_{g}^{i}}(x)$ that provides the membership of the object *x* in the subset ${S_{g}^{i}}$, where ${f_{g}^{i}}(x)=1$ if $x\in {S^{i}_{g}}$, ${f_{g}^{i}}(x)=0$ if $x\notin {S^{i}_{g}}$, and ${f_{g}^{i}}(x) \in (0,1)$ if *x* has a fuzzy membership in ${S^{i}_{g}}$, i.e. ${f_{g}^{i}}(x)$ can be seen as the probability of *x* being in ${S^{i}_{g}}$. Furthermore, let the segmentation, being evaluated, be represented by the partition $S_{t}=\left \{{S_{t}^{1}},{S_{t}^{2}}\right \}$ of *X* with the assignment function ${f_{t}^{j}}(x)$ that provides the membership of *x* in the class ${S_{t}^{j}}$, defined analogously to ${f_{g}^{i}}$. Note that in this paper, we only deal with partitions with two classes, namely the class of interest (anatomy or feature) and the background. We always assume that the first class $\left ({S_{g}^{1}}, {S_{t}^{1}}\right)$ is the class of interest and the second class $\left ({S_{g}^{2}}, {S_{t}^{2}}\right)$ is the background. The assignment functions ${f_{g}^{i}}$ and ${f_{t}^{j}}$ can either be crisp when their range is {0,1} or fuzzy when their range is [0,1]. Note that the crisp partition is just a special case of the fuzzy partition. We also assume that the memberships of a given point *x* always sum to one over all classes. This implies that ${f_{g}^{1}}(x)+{f_{g}^{2}}(x)=1$ and ${f_{t}^{1}}(x)+{f_{t}^{2}}(x)=1$ for all *x*∈*X*. In the remainder of this section, we define the foundation of methods and algorithms used to compute all the metrics presented in Table 1. We structure the discussion in this section to follow the metric grouping given in the column “category”. This provides a structure that is advantageous for the implementation of the evaluation tool, that is to improve the efficiency by making use of the synergy between the metrics in each group to avoid repeated calculation of the same parameters.

#### Spatial overlap based metrics

In the following subsections, the overlap based metrics are defined. Because all metrics from this category can be derived from the four basic cardinalities of the so-called confusion matrix, namely the true positives (*TP*), the false positives (*FP*), the true negatives (*TN*), and the false negatives(*FN*), we define these cardinalities for crisp as well as fuzzy segmentations, then we define the metrics based on them.

**Basic cardinalities** For two crisp partitions (segmentations) *S*_*g*_ and *S*_*t*_, the confusion matrix consists of the four common cardinalities that reflect the overlap between the two partitions, namely *TP*, *FP*, *FN*, and *TN*. These cardinalities provide for each pair of subsets *i*∈*S*_*g*_ and *j*∈*S*_*t*_ the sum of agreement *m*_*ij*_ between them. That is

(1) $$ m_{ij}= \sum\limits_{r=1}^{|X|}\, {f_{g}^{i}}(x_{r}) {f_{t}^{j}}(x_{r})   $$

where *TP*=*m*_11_, *FP*=*m*_10_, *FN*=*m*_01_, and *TN*=*m*_00_. Table 2 shows the confusion matrix of the partitions *S*_*g*_ and *S*_*t*_. Note that Eq. 1 assumes crisp memberships. In the next section the four cardinalities are generalized to fuzzy partitions.

**Table 2:** Confusion matrix: comparing ground truth segmentation *S*_*g*_ with test segmentation *S*_*t*_. Confusion matrix: comparing ground truth segmentation *S*_*g*_ with test segmentation *S*_*t*_

Subset	${S_{t}^{1}}$	${S_{t}^{2}}(=\overline {{S_{t}^{1}}})$
${S_{g}^{1}}$	*T* *P*(*m*_11_)	*F* *P*(*m*_12_)
${S_{g}^{2}}(=\overline {{S_{g}^{1}}})$	*F* *N*(*m*_21_)	*T* *N*(*m*_22_)

**Generalization to fuzzy segmentations:** Intuitively, one favorable way to generalize the overlap based metrics presented in Table 1 for fuzzy partitions is to provide a method for calculating the cardinalities of the confusion matrix for fuzzy sets because the confusion matrix is the base on which all metrics in this category are defined. To this end, the main task is to calculate the agreement between two segmentations, where the assignments of voxels to segments are probabilities (fuzzy). It is common for this purpose to use a suitable triangular norm (t-norm) to calculate the agreement between two fuzzy assignments [11, 12]. Given two probabilities *p*1 and *p*2 representing the memberships of a particular element (voxel) to a particular class (segment) according to two different classifiers (segmenters), we use the *min*(*p*1,*p*2) as a t-norm as the agreement between the two classifiers. That is, we define the agreement function *g*:[0,1]×[0,1]→[0,1] that models the agreement on a particular voxel being assigned to a particular segment as *g*(*p*1,*p*2)=*min*(*p*1,*p*2). This also means that the agreement on the same voxel being assigned to the background is given by *g*(1−*p*1,1−*p*2). Intuitively, the disagreement between the segmenters is the difference between the probabilities given by |*p*1−*p*2|. However, since the comparison is asymmetrical (i.e. one of the segmentations is the ground truth and the other is the test segmentation), we consider the signed difference rather than the absolute difference as in Eqs. 3 and 5. The four cardinalities defined in Eq. 1 can be now generalized to the fuzzy case as follows:

(2) $$ TP = \sum\limits_{r=1}^{|X|} min\left({f_{t}^{1}}(x_{r}),{f_{g}^{1}}(x_{r})\right)   $$

(3) $$ FP = \sum\limits_{r=1}^{|X|} max\left({f_{t}^{1}}(x_{r})-{f_{g}^{1}}(x_{r}),0\right)   $$

(4) $$ TN = \sum\limits_{r=1}^{|X|} min\left({f_{t}^{2}}(x_{r}), {f_{g}^{2}}(x_{r})\right)   $$

(5) $$ FN = \sum\limits_{r=1}^{|X|} max\left({f_{t}^{2}}(x_{r})-{f_{g}^{2}}(x_{r}),0\right)   $$

Note that in Eqs. 2 to 5, ${f_{g}^{i}}(x_{t})$ and ${f_{t}^{j}}(x_{t})$ are used in place of *p*1 and *p*2 since each of the functions provides the probability of the membership of a given point in the corresponding segment, and in the special case of crisp segmentation, they provide 0 and 1.

Other norms have been used to measure the agreement between fuzzy memberships like the product t-norm, the L-norms, and the cosine similarity. We justify using the min t-norm by the fact that, in contrast to the other norms, the min t-norm ensures that the four cardinalities, calculated in Eqs. 2 to 5, sum to the total number of voxels, i.e. *TP*+*FP*+*TN*+*FN*=|*X*| which is an important requirement for the definition of metrics.

**Calculation of overlap based metrics** In this section, we define each of the overlap based metrics in Table 1 based on the basic cardinalities in Eq. 1 (crisp) or Eqs. 2 to 5 (fuzzy).

The Dice coefficient [13] (*DICE*), also called the overlap index, is the most used metric in validating medical volume segmentations. In addition to the direct comparison between automatic and ground truth segmentations, it is common to use the *DICE* to measure reproducibility (repeatability). Zou et al. [1] used the *DICE* as a measure of the reproducibility as a statistical validation of manual annotation where segmenters repeatedly annotated the same MRI image, then the pair-wise overlap of the repeated segmentations is calculated using the *DICE*, which is defined by

(6) $$ DICE = \frac{2\left|{S_{g}^{1}} \cap {S_{t}^{1}}\right|}{\left|{S_{g}^{1}}\right| + \left|{S_{t}^{1}}\right|} = \frac{2 TP}{2 TP + FP + FN}   $$

The Jaccard index (*JAC*) [14] between two sets is defined as the intersection between them divided by their union, that is

(7) $$ JAC = \frac{\left|{S_{g}^{1}} \cap {S_{t}^{1}}\right|}{\left|{S_{g}^{1}} \cup {S_{t}^{1}}\right|} = \frac{TP}{TP + FP + FN}   $$

We note that *JAC* is always larger than *DICE* except at the extrema {0,1} where they are equal. Furthermore the two metrics are related according to

(8) $$ \begin{aligned} JAC&=\frac{\left|{S_{g}^{1}} \cap {S_{t}^{1}}\right|}{\left|{S_{g}^{1}} \cup {S_{t}^{1}}\right|}=\frac{2\left|{S_{g}^{1}} \cap {S_{t}^{1}}\right|}{2\left(\left|{S_{g}^{1}}\right|+\left|{S_{t}^{1}}\right|- \left|{S_{g}^{1}} \cap {S_{t}^{1}}\right|\right)}\\ &=\frac{DICE}{2-DICE} \end{aligned}   $$

Similarly, one can show that

(9) $$ DICE=\frac{2JAC}{1+JAC}   $$

That means that both of the metrics measure the same aspects and provide the same system ranking. Therefore, it does not provide additional information to select both of them together as validation metrics as done in [15–17].

True Positive Rate (*TPR*), also called Sensitivity and Recall, measures the portion of positive voxels in the ground truth that are also identified as positive by the segmentation being evaluated. Analogously, True Negative Rate (*TNR*), also called Specificity, measures the portion of negative voxels (background) in the ground truth segmentation that are also identified as negative by the segmentation being evaluated. However these two measures are not common as evaluation measures of medical image segmentation because of their sensibility to segments size, i.e. they penalize errors in small segments more than in large segments [6, 8, 10]. Note that the terms positive and negative are rather for crisp segmentation. However, the generalization in Eqs. 2 to 5 extends the meaning of the terms to grade agreement. These two measures are defined as follows:

(10) $$ Recall=Sensitivity=TPR=\frac{TP}{TP+FN}   $$

(11) $$ Specificity=TNR =\frac{TN}{TN+FP}   $$

There are two other measures that are related to these metrics, namely the false positive rate (*FPR*), also called Fallout, and the false negative rate (*FNR*). They are defined by

(12) $$ Fallout=FPR=\frac{FP}{FP+TN}=1-TNR   $$

(13) $$ FNR=\frac{FN}{FN+TP}=1-TPR   $$

The equivalence in Eqs. 12 and 13 implies that only one of each two equivalent measures should be selected for validation and not both of them together [10], i.e. either *FPR* or *TNR* and analogously, either *FNR* or *TPR*. Another related measure is the precision, also called the positive predictive value (*PPV*) which is not commonly used in validation of medical images, but it is used to calculate the F-Measure. It is defined by

(14) $$ Precision=PPV= \frac{TP}{TP+FP}   $$

*F*_*β*_-*Measure* (*FMS*_*β*_) was firstly introduced in [18] as an evaluation measure for information retrieval. However, it is a special case of the Rijsbergen’s effectiveness measure^3^ introduced in [19]. *F*_*β*_-*Measure* is a trade-off between *PPV* (precision, defined in Eq. 14) and *TPR* (recall, defined in Eq. 10). *F*_*β*_-*Measure* is given by

(15) $$ FMS_{\beta}=\frac{(\beta^{2} +1)\cdot PPV \cdot TPR}{\beta^{2} \cdot PPV+TPR}   $$

With *β*=1.0 (precision and recall are equally important), we get the special case *F*_1_-*Measure* (*FMS*_1_); we call it *FMS* for simplicity. It is also called the harmonic mean and given by

(16) $$ FMS=\frac{2\cdot PPV \cdot TPR}{PPV+TPR}   $$

Here, we note that the *FMS* is mathematically equivalent to *DICE*. This follows from a trivial substitution for *TPR* and *PPV* in Eq. 16 by their values from Eqs. 10 and 14. After simplification it results in the definition of *DICE* (Eq. 6).

The global consistency error (*GCE*) [20] is an error measure between two segmentations. Let *R*(*S*,*x*) be defined as the set of all voxels that reside in the same region of segmentation *S* where the voxel *x* resides. For the two segmentations *S*1 and *S*2, the error at voxel *x*, *E*(*S*1,*S*2,*x*) is defined as

(17) $$ E(S_{t},S_{g},x) = \frac{|R(S_{t},x)\backslash R(S_{g},x)|}{|R(S_{t},x)|}   $$

Note that *E* is not symmetric. The global consistency error (GCE) is defined as the error averaged over all voxels and is given by

(18) $$  \begin{aligned} GCE(S_{t},S_{g}) = \frac{1}{n} min \left\{ {\sum_{i}^{n}} E(S_{t},S_{g},x_{i}), {\sum_{i}^{n}} E(S_{g},S_{t},x_{i})\right\} \end{aligned}   $$

Eq. 18 can be expressed in terms of the four cardinalities defined in Eqs. 2 to 4 to get the *GCE* between the (fuzzy) segmentations *S*_*g*_ and *S*_*t*_ as follows

(19) $$ \begin{aligned} GCE =&\, \frac{1}{n} min \left\{\frac{FN(FN+2 TP)}{TP+FN}+\frac{FP(FP+2 TN)}{TN+FP},\right.\\ &\left.\frac{FP(FP+2 TP)}{TP+FP}+\frac{FN(FN+2 TN)}{TN+FN} \right\} \end{aligned}   $$

**Overlap measures for multiple labels** All the overlap measures presented previously assume segmentations with only one label. However, it is common to compare segmentations with multiple labels, e.g. two-label tumor segmentation (core and edema). Obviously, one way is to compare each label separately using the overlap measures presented previously, but this would lead to the problem of how to average the individual similarities to get a singly score. For this evaluation tool, we use the overlap measures proposed by Crum et al. [17], namely *DICE*_*ml*_ and *JAC*_*ml*_ which are generalized to segmentations with multiple labels. For the segmentations *A* and *B*

(20) $$ JAC_{ml} = \frac{\sum\limits_{labels,l} \alpha l \sum\limits_{voxels, i} MIN (A_{li}, B_{li})} {\sum\limits_{labels,l} \alpha l \sum\limits_{voxels, i} MAX (A_{li}, B_{li})}   $$

where *A*_*li*_ is the value of voxel *i* for label *l* in segmentation *A* (analogously for *B*_*li*_) and *αl* is a label-specific weighting factor that affects how much each label contributes to the overlap accumulated over all labels. Here, the *MIN*(.) and *MAX*(.) are the norms used to represent the intersection and union in the fuzzy case. *DICE*_*ml*_ can be then calculated from *JAC* according to Eq. 9, i.e. *DICE*_*ml*_=2*JAC*_*ml*_/(1+*JAC*_*ml*_). Note that the equations above assume the general case of multiple label and fuzzy segmentation. However, in multiple label segmentations, voxels values mostly represent the labels (classes) rather than probabilities which means in most available image formats, there are either multiple label or fuzzy segmentations.

#### Volume based metrics

As the name implies, volumetric similarity (*VS*) is a measure that considers the volumes of the segments to indicate similarity. There is more than one definition for the volumetric distance in the literature, however we consider the definition in [21–23] and [15], namely the absolute volume difference divided by the sum of the compared volumes. We define the Volumetric Similarity (*VS*) as 1−*VD* where *VD* is the volumetric distance. That is

(21) $$ VS=1-\frac{||{S_{t}^{1}}|-|{S_{g}^{1}}||}{|{S_{t}^{1}}|+|{S_{g}^{1}}|}= 1-\frac{|FN-FP|}{2 TP+FP+FN}   $$

Note that although the volume similarity is define using the four cardinalities, it is not considered an overlap-based metric, since here the absolute volume of the segmented region in one segmentation is compared with the corresponding volume in the other segmentation. This means that the overlap between the segments is absolutely not considered. Actually, the volumetric similarity can have its maximum value even when the overlap is zero. More details in Section “Metric analysis”.

#### Pair counting based metrics

In this section, pair-counting based metrics, namely the Rand index and its extensions, are defined. At first we define the four basic pair-counting cardinalities, namely *a*, *b*, *c*, and *d* for crisp and fuzzy segmentations and then we define the metrics based on these cardinalities.

**Basic cardinalities** Given two partitions of the point set *X* being compared, let *P* be the set of $\binom {n}{2}$ tuples that represent all possible object pairs in *X*×*X*. These tuples can be grouped into four categories depending on where the objects of each pair are placed according to each of the partitions. That is, each tuple (*x*_*i*_,*x*_*j*_)∈*P* is assigned to one of four groups whose cardinalities are *a*, *b*, *c*, and *d*.

- Group I: if *x*_*i*_ and *x*_*j*_ are placed in the same subset in both partitions *S*_*g*_ and *S*_*t*_. We define *a* as the cardinality of Group I.
- Group II: if *x*_*i*_ and *x*_*j*_ are placed in the same subset in *S*_*g*_ but in different subsets in *S*_*t*_. We define *b* as the cardinality of Group II.
- Group III: if *x*_*i*_ and *x*_*j*_ are placed in the same subset in *S*_*t*_ but in different subsets in *S*_*g*_. We define *c* as the cardinality of Group III.
- Group IV: if *x*_*i*_ and *x*_*j*_ are placed in different subsets in both partitions *S*_*g*_ and *S*_*t*_. We define *d* as the cardinality of Group IV.

Note that the count of tuples in Groups I and IV represents the agreement (*a*+*d*) whereas the count of tuples in Groups II and III (*b*+*c*) represents the disagreement between the two partitions.

Obviously, because there are $\binom {n}{2}=n(n-1)/2$ tuples, the direct calculation of these parameters needs *O*(*n*^2^) runtime. However, Brennan and Light [24] showed that these cardinalities can be calculated using the values of the confusion matrix without trying all pairs and thus avoiding the *O*(*n*^2^) complexity, that is

(22) $$ a = \frac{1}{2}\sum_{i=1}^{r} \sum_{j=1}^{s} m_{ij}(m_{ij}-1)   $$

(23) $$ b = \frac{1}{2}\left(\sum_{j=1}^{s} m_{.j}^{2} - \sum_{i=1}^{r} \sum_{j=1}^{s} m_{ij}^{2} \right)   $$

(24) $$ c = \frac{1}{2} \left(\sum_{j=1}^{r} m_{i.}^{2} - \sum_{i=1}^{r} \sum_{j=1}^{s} m_{ij}^{2} \right)   $$

(25) $$ d = n(n-1)/2 - (a+b+c)   $$

where *r* and *s* are the class counts in the compared partitions, *m*_*ij*_ is the confusion matrix (Table 2), *m*_*i*._ denotes the sum over the *i*th row, and *m*_.*j*_ denotes the sum over the *j*th column. Note that here, in contrast to the overlap based metrics, there is no restriction on the number of classes in the compared partitions. However, in the proposed evaluation tool, we are interested in segmentations with only two classes, namely the anatomy and the background; i.e. *r*=*s*=2. We define the four cardinalities for this special case, more specifically for the segmentations *S*_*g*_ and *S*_*t*_ defined in Section “Metric definitions and Algorithms” based on the four overlap parameters defined in Section “Basic cardinalities”

(26) $$ \begin{aligned} a &= \frac{1}{2}\left[TP(TP-1)+FP(FP-1)\right.\\ &\left.+TN(TN-1)+FN(FN-1)\right]  \end{aligned}  $$

(27) $$ \begin{aligned} b &= \frac{1}{2}\left[(TP+FN)^{2}+(TN+FP)^{2}\right.\\ &\left.\quad-(TP^{2}+TN^{2}+FP^{2}+FN^{2})\right] \end{aligned}   $$

(28) $$ \begin{aligned} c &= \frac{1}{2}\left[(TP+FP)^{2}+(TN+FN)^{2}\right.\\ &\left.\quad-(TP^{2}+TN^{2}+FP^{2}+FN^{2})\right] \end{aligned}   $$

(29) $$ d = n(n-1)/2 - (a+b+c)   $$

**Generalization to fuzzy segmentations** As mentioned above, since the cardinalities *a*, *b*, *c*, and *d* are by definition based on grouping all the pairwise tuples defined on *S*_*g*_ and *S*_*t*_, this requires processing *n*(*n*−1)/2 tuples which means a direct computation of these cardinalities for fuzzy segmentations takes *O*(*n*^2^) runtime. For medical segmentation, this complexity could be a problem since the size of medical volumes could reach 8-digit numbers. Methods (Huellermeier et al. [25], Brouwer [26], Campello [12]) have been proposed that calculate the Rand index and its extension for fuzzy segmentations using different approaches. None of these approaches is efficiently applicable in the 3D medical imaging domain because they all have a run time complexity of *O*(*n*^2^). However, Anderson et al. [27] proposed a method that calculates the four cardinalities for fuzzy sets in *O*(*n*) runtime. This is achieved by combining two already known strategies: (i) calculating the confusion matrix for fuzzy sets using some agreement function e.g. Eqs. 2 to 5 and (ii) calculating the four cardinalities by applying Eqs. 22 to 25 on the values of the fuzzy confusion matrix calculated in (i). This approach is used in this paper which means that Eqs. 26 to 29 already provide the fuzzy cardinalities according to [27], given the parameters *TP*, *FP*, *TN* and *FN* are calculated for fuzzy sets. In the next subsection, the Rand index and the adjusted rand index are calculated based on these cardinalities.

**Calculation of pair-counting based metrics** The Rand Index (*RI*), proposed by W. Rand [28] is a measure of similarity between clusterings. One of its important properties is that it is not based on labels and thus can be used to evaluate clusterings as well as classifications. The RI between two segmentations *S*_*g*_ and *S*_*t*_ is defined as

(30) $$ RI(S_{g},S_{t}) = \frac{a+b}{a+b+c+d}   $$

where *a*, *b*, *c*, *d* are the cardinalities defined in Eqs. 26 to 29.

The Adjusted Rand Index (*ARI*), proposed by Hubert and Arabie [29], is a modification of the Rand Index that considers a correction for chance. It is given by

(31) $$ ARI = \frac{\sum\limits_{ij} \binom{m_{ij}}{2} - \sum\limits_{i}\binom{m_{i.}}{2} \sum\limits_{j}\binom{m_{.j}}{2}/\binom{n}{2}}{\frac{1}{2}\left[\sum\limits_{i}\binom{m_{i.}}{2}+\sum\limits_{j}\binom{m_{.j}}{2}\right] - \sum\limits_{i}\binom{m_{i.}}{2}\sum\limits_{j}\binom{m_{.j}}{2}/\binom{n}{2}}   $$

where *n* is the object count, *m*_*ij*_ is the confusion matrix (Table 2), *m*_*i*._ denotes the sum over the *i*th row, and *m*_.*j*_ denotes the sum over the *j*th column. The *ARI* can be expressed by the four cardinalities as

(32) $$ ARI = \frac{2(a d - b c)}{c^{2} + b^{2} + 2 a d+ (a+d) (c+b)}   $$

#### Information theoretic based metrics

The Mutual Information (*MI*) between two variables is a measure of the amount of information one variable has about the other. Or in other words, the reduction in uncertainty of one variable, given that the other is known [30]. It was firstly used as a measure of similarity between images by Viola and Wells [31]. Later, Russakoff et al. [32] used the *MI* as a similarity measure between image segmentations; in particular, they calculate the *MI* based on regions (segments) instead of individual pixels. The *MI* is related to the marginal entropy *H*(*S*) and the joint entropy *H*(*S*_1_,*S*_2_) between images defined as

(33) $$ H(S) = -\sum_{i} p(S^{i})~log~p(S^{i})   $$

(34) $$ H(S_{1},S_{2}) = -\sum_{ij} p\left({S_{1}^{i}},{S_{2}^{j}}\right)~log~p\left({S_{1}^{i}},{S_{2}^{j}}\right)   $$

where *p*(*x*,*y*) is joint probability, *S*^*i*^ are the regions (segments) in the image segmentations and *p*(*S*^*i*^) are the probabilities of these regions that can be expressed in terms of the four cardinalities *TP*, *FP*, *TN* and *FN*, which are calculated for the fuzzy segmentations (*S*_*g*_ and *S*_*t*_) in Eqs. 2 to 5 as follows

(35) $$ \begin{aligned} p\left({S_{g}^{1}}\right)&=(TP+FN)/n\\ p\left({S_{g}^{2}}\right)&=(TN+FN)/n\\ p\left({S_{t}^{1}}\right)&=(TP+FP)/n\\ p\left({S_{t}^{2}}\right)&=(TN+FP)/n \end{aligned}   $$

where *n*=*TP*+*FP*+*TN*+*FN* is the total number of voxels. Because *TP*, *TN*, *FP* and *FN* are by definition cardinalities of disjoint sets that partition the volume, the joint probabilities are given by

(36) $$ p\left({S_{1}^{i}}, {S_{2}^{j}}\right)= \frac{\left|{S_{1}^{i}} \cap {S_{2}^{j}}\right|}{n}   $$

which implies

(37) $$ \begin{aligned} p\left({S_{1}^{1}}, {S_{2}^{1}}\right)&= \frac{TP}{n}\\ p\left({S_{1}^{1}}, {S_{2}^{2}}\right)&= \frac{FN}{n}\\ p\left({S_{1}^{2}}, {S_{2}^{1}}\right)&= \frac{FP}{n}\\ p\left({S_{1}^{2}}, {S_{2}^{2}}\right)&= \frac{TN}{n}\\ \end{aligned}   $$

The MI is then defined as

(38) $$ MI(S_{g},S_{t}) = H(S_{g}) + H(S_{t}) - H(S_{g},S_{t})   $$

The Variation of Information (*VOI*) measures the amount of information lost (or gained) when changing from one variable to the other. Marin [33] first introduced the *VOI* measure for comparing clusterings partitions. The *VOI* is defined using the entropy and mutual information as

(39) $$ VOI(S_{g},S_{t}) = H(S_{g}) + H(S_{t}) - 2~MI(S_{g},S_{t})   $$

#### Probabilistic metrics

The Interclass Correlation (*ICC*) [34] is a measure of correlations between pairs of observations that don’t necessarily have an order, or are not obviously labeled. It is common to use the *ICC* as a measure of conformity among observers; in our case it is used as a measure of consistency between two segmentations. *ICC* is given by

(40) $$ ICC = \frac{{\sigma_{S}^{2}} }{{\sigma_{S}^{2}}+\sigma_{\epsilon}^{2}}   $$

where *σ*_*S*_ denotes variance caused by differences between the segmentations and *σ*_*ε*_ denotes variance caused by differences between the points in the segmentations [34]. Applied to the segmentations *S*_*g*_ and *S*_*t*_, *ICC* is defined as

(41) $$ \begin{aligned} ICC &= \frac{MS_{b} - MS_{w}}{MS_{b} + (k-1) MS_{w}} ~~~with\\ MS_{b}&= \frac{2}{n-1}\sum\limits_{x} (m(x)-\mu)^{2}\\ MS_{w}&=\frac{1}{n}\sum\limits_{x} \left(\,f_{g}(x)-m(x)\right)^{2}+\left(\,f_{t}(x)-m(x)\right)^{2} \end{aligned}   $$

where *MS*_*b*_ denotes the mean squares between the segmentations (called between group MS), *MS*_*w*_ denotes the mean squares within the segmentations (called within group MS), *k* is the number of observers which is 2 in case of comparing two segmentations, *μ* is the grand mean, i.e. the mean of the means of the two segmentations, and *m*(*x*)=(*f*_*g*_(*x*)+*f*_*t*_(*x*))/2 is the mean at voxel *x*.

The Probabilistic Distance (*PBD*) was developed by Gerig et al. [8] as a measure of distance between fuzzy segmentations. Given two fuzzy segmentations, *A* and *B*, then the PBD is defined by

(42) $$ PBD(A,B) = \frac{\int |P_{A} - P_{B}|}{2\int P_{AB}}   $$

where *P*_*A*_(*x*) and *P*_*B*_(*x*) are the probability distributions representing the segmentations and *P*_*AB*_ is their pooled joint probability distribution. Applied on *S*_*g*_ and *S*_*t*_, defined in Section “Metric definitions and Algorithms”, the PBD is defined as

(43) $$ PBD(S_{g},S_{t}) = \frac{\sum\limits_{x} |\,f_{g}(x) - f_{t}(x)|}{2\sum\limits_{x} f_{g}(x)f_{t}(x)}   $$

The Cohen Kappa Coefficient (*KAP*), proposed in [35], is a measure of agreement between two samples. As an advantage over other measures, *KAP* takes into account the agreement caused by chance, which makes it more robust. *KAP* is given by

(44) $$ KAP = \frac{P_{a}-P_{c}}{1-P_{c}}   $$

where *P*_*a*_ is the agreement between the samples and *P*_*c*_ is the hypothetical probability of chance agreement. The same can be expressed in form of frequencies to facilitate the computation as follows

(45) $$ KAP = \frac{f_{a}-f_{c}}{N-f_{c}}   $$

where *N* is the total number of observations, in our case the voxels. The terms in Eq. 45 can be expressed in terms of the four overlap cardinalities, calculated for fuzzy segmentations (Eqs. 2 to 5), to get

(46) $$ \begin{aligned} f_{a} &= TP+TN \\ f_{c} &= \frac{(TN+FN)(TN+FP)+(FP+TP)(FN+TP)}{N}  \end{aligned}  $$

The *ROC* curve (Receiver Operating Characteristic) is the plot of the true positive rate (*TPR*) against the false positive rate (*FPR*). The area under the *ROC* curve (*AUC*) was first presented by Hanley and McNeil [36] as a measure of accuracy in the diagnostic radiology. Later, Bradley [37] investigated its use in validating machine learning algorithms. The *ROC* curve, as a plot of *TPR* against *FPR*, normally assumes more than one measurement. For the case where a test segmentation is compared to a ground truth segmentation (one measurement), we consider a definition of the *AUC* according to [38], namely the area of the trapezoid defined by the measurement point and the lines *TPR*=0 and *FPR*=1, which is given by

(47) $$ \begin{aligned} AUC &= 1-\frac{FPR+FNR}{2}\\ &= 1-\frac{1}{2}\left(\frac{FP}{FP+TN}+\frac{FN}{FN+TP}\right) \end{aligned}   $$

#### Spatial distance based metrics

Spatial distance based metrics are widely used in the evaluation of image segmentation as dissimilarity measures. They are recommended when the segmentation overall accuracy, e.g the boundary delineation (contour), of the segmentation is of importance [6]. As the only category in this paper, distance-based measures take into consideration the spatial position of voxels. More about the properties of distance metrics is in Section “Results and discussion”. In this section, we present three distance metrics, namely the Hausdorff distance, the Average distance and the Mahalanobis distance. All distances calculated in this section are in voxel, which means the voxel size is not taken into account.

**Distance between crisp volumes** The Hausdorff Distance (*HD*) between two finite point sets *A* and *B* is defined by

(48) $$ HD(A,B) = max (h(A,B), h(B,A))   $$

where *h*(*A*,*B*) is called the directed Hausdorff distance and given by

(49) $$ h(A,B) = \max \limits_{a \in A} \min\limits_{b \in B} \|a-b\|   $$

where ∥*a*−*b*∥ is some norm, e.g. Euclidean distance. An algorithm that directly calculates the *HD* according to Eq. 49 takes an execution time of *O*(|*A*||*B*|). There are many algorithms that calculate the *HD* with lower complexity. In this paper, we use the algorithm proposed in [39] which calculates the *HD* in a nearly-linear time complexity.

The *HD* is generally sensitive to outliers. Because noise and outliers are common in medical segmentations, it is not recommended to use the *HD* directly [8, 40]. However, the quantile method proposed by Huttenlocher et al. [41] is one way to handle outliers. According to the Hausdorff quantile method, the *HD* is defined to be the *q*^*th*^ quantile of distances instead of the maximum, so that possible outliers are excluded, where *q* is selected depending on the application and the nature of the measured point sets.

The Average Distance, or the Average Hausdorff Distance (*AVD*), is the *HD* averaged over all points. The *AVD* is known to be stable and less sensitive to outliers than the *HD*. It is defined by

(50) $$ AVD(A,B) = max (d(A,B), d(B,A))   $$

where *d*(*A*,*B*) is the directed Average Hausdorff distance that is given by

(51) $$ d(A,B) = \frac{1}{N}\sum\limits_{a \in A} \min\limits_{b \in B} \|a-b\|   $$

To efficiently calculate the *AVD* and avoid a complexity of *O*(|*A*||*B*|) (scanning all possible point pairs), we use a modified version of the nearest neighbor (NN) algorithm proposed by Zhao et al. [42] in which a 3D cell grid is built on the point cloud and for each query point, a search subspace (a subset of the cell grids that contains the nearest neighbor) is found to limit the search and reduce the number of distance calculations needed. We added three modifications to this algorithm that make use of the nature of segmentations, namely that they are mostly dense point clouds. These modifications enable efficiently finding the exact NN. In the first modification, when calculating the pairwise distances from segment *A* to *B*, we remove the intersection *A*∩*B* from consideration because here all the distances are zero, that is we calculate only *A*∖*B* to *B*. For the second modification, instead of considering all points of *B*, we consider only the points on the surface of segment *B*. This is justified by the fact that when moving in a line from a point in segment *A* (but not in the intersection) to the segment *B*, the first point crossed in *B* is on the surface and this is the shortest distance, which means all points inside the segments are not relevant. The third modification is to find the radius *r* that defines a convenient search subspace for a given query point *q*∈*A*. We find *r* by moving from *q* to the mean of *B* and if a point *p*∈*B* is crossed, we define *r* as the distance between *q* and *p*, i.e. the search subspace consists of all cell grids contained in or crossed by the sphere centered on *q* with radius *r*. If no point *p* is found (which is unlikely to happen with segmentations), an exhaustive search is performed.

The Mahalanobis Distance (*MHD*) [43] between two points in a point cloud, in contrast to the Euclidean distance, takes into account the correlation of all points in the point cloud containing the two points. The *MHD* between the points *x* and *y* in the point cloud *A* is given by

(52) $$ MHD(x,y) = \sqrt{(x-y)^{T}S^{-1}(x-y)}   $$

where *S*^−1^ is the inverse of the covariance matrix *S* of the point cloud and the superscript *T* denotes the matrix transpose. Note that *x* and *y* are two points in the same point cloud, but in the validation of image segmentation, two point clouds are compared. For this task, we use the variant of *MHD* according to G. J. McLachlan [44], where the *MHD* is calculated between the means of the compared point clouds and the common covariance matrix of them is considered as *S*. Hence the Mahalanobis distance *MHD*(*X*,*Y*) between the point sets *X* and *Y* is

(53) $$ MHD(X,Y) = \sqrt{(\mu_{x}-\mu_{y})^{T}S^{-1}(\mu_{x}-\mu_{y})}   $$

where *μ*_*x*_ and *μ*_*y*_ are the means of the point sets and the common covariance matrix of the two sets is given by

(54) $$ S = \frac{n_{1} S_{1} + n_{2} S_{2}}{n_{1}+n_{2}}   $$

where *S*_1_, *S*_2_ are the covariance matrices of the voxel sets and *n*_1_, *n*_2_ are the numbers of voxels in each set.

**Extending the distances to fuzzy volumes** Different approaches have been proposed to measure the spatial distance between fuzzy images. The approaches described in [45] are based on defuzzification (finding a crisp representation) either by minimizing the feature distance, which leads to the problem of selecting the features, or by finding crisp representations with a higher resolution which leads to multiplication of the grid dimensions and therefore negatively impacts the efficiency of time consuming algorithms, like *HD* and *AVD*. For this evaluation tool, we use a discrete form of the approach proposed in [46] i.e. the average of distances at different *α*-cuttings depending on a given number of cutting levels *k*. The *HD* distance between the fuzzy segmentations *A* and *B* is thus given by

(55) $$ \overline{HD}_{k}(A,B) = \frac{1}{k} \sum\limits_{i=1}^{k} HD_{\frac{i}{k}}(A,B)   $$

(56) $$ HD_{\alpha}(A,B) = HD(A_{\alpha}, B_{\alpha})   $$

where *A*_*α*_ and *B*_*α*_ are the crisp representations resulting from thresholding the fuzzy volumes *A* and *B* at cutting level *α*, *HD*_*α*_ is the *HD* at cutting level *α*, and *k*>0 is an integer that gives the number of cutting levels considered.

Analogously, the *AVD* and *MHD* between the fuzzy volumes *A* and *B* are given by

(57) $$ \overline{AVD}_{k}(A,B) = \frac{1}{k}\sum\limits_{i=1}^{k} AVD\left(A_{\frac{i}{k}}, B_{\frac{i}{k}}\right)   $$

(58) $$ \overline{MHD}_{k}(A,B) = \frac{1}{k}\sum\limits_{i=1}^{k} MHD\left(A_{\frac{i}{k}}, B_{\frac{i}{k}}\right)   $$

If the parameters *k* and *α* are omitted, i.e. *HD*, *AVD* and *MHD*, we assume distances at the cutting level *α*=0.5.

### Multiple definition of metrics in the literature

We present three examples representing three categories of inconsistency in the literature regarding the definition of the metrics to underline the need of a standardization of evaluation metrics and motivate a standard evaluation tool for medical segmentations. The first category is caused by misinterpretation resulting in misleading definitions, for example the confusion of the pair counting cardinalities (*a*, *b*, *c* and *d*) with the overlap cardinalities (*TP*, *FP*, *TN* and *FN*). In some papers [12, 25, 27, 47], the pair-counting cardinalities are used in place of the overlap cardinalities although they are mathematically and semantically different. According to the definition, the pair-counting cardinalities result from grouping *n*(*n*−1)/2 tuples defined on *X*×*X* (Section “Basic cardinalities”) whereas the overlap-based cardinalities (Section “Basic cardinalities”) result from the class overlap i.e. pairwise comparison of *n* voxel assignments. In the papers mentioned above, several overlap-based metrics including the Jaccard index are defined using the pair-counting cardinalities in place of the overlap cardinalities. To illustrate how strongly the results differ in the two cases, we show examples in Table 3. In each example, the partitions *P*1 and *P*2 are compared using the Jaccard index which is calculated in two ways: the first (*JAC*_1_) using the overlap cardinalities according to [14] and [48], the second (*JAC*_2_) using the pair counting cardinalities according to [25, 27, 47] and [12]. The values are different except in the first example.

**Table 3:** Pair counting cardinalities versus overlap cardinalities in examples. Five examples show that the pair counting cardinalities (*a*, *b*, *c*, and *d*) cannot be used in place of the overlap cardinalities (*TP*, *FP*, *FN*, and *TN*) to calculate the Jaccard index, as it is commonly used in the literature

P1	P2	*TP*	*FP*	*FN*	*TN*	*J* *A* *C*_1_	a	b	c	d	*J* *A* *C*_2_
1,0,1,1	1,1,0,0	1	2	1	0	0.25	1	2	1	2	0.25
1,1,1,1	0,0,0,1	1	3	0	0	0.25	3	3	0	0	0.5
0,1,0,1	1,1,0,0	1	1	1	1	0.33	0	2	2	2	0.0
0,0,0,0	0,0,0,1	0	0	1	3	0.0	3	0	3	0	0.5
1,0,0,1	1,1,0,1	2	0	1	1	0.67	1	2	1	2	0.25

The second category is naming inconsistency, where the same name is used to denote two different metrics. One example is the volumetric similarity (*VS*). While *VS* is defined in [21–23] and [15] as the absolute volume difference divided by the sum of the compared volumes (Eq. 21), there is another metric definition under the same name in [49] defined as twice the volume of the intersection divided by the volume sum in percent, i.e.

(59) $$ VS=2\frac{|S_{t}\cap S_{g}|}{|S_{t} + S_{g}|}.100\, \%   $$

The last category is the multiple definition that stems from different theoretical approaches for estimating the same value. For example, the Interclass Correlation (*ICC*) has an early definition proposed by Fisher [50]. Later, several estimators of the *ICC* have been proposed, one of them is the definition in Eq. 40 proposed by Shrout and Fleiss [34]. Note that although these definitions are totally different, in contrast to the second category, they all aim to estimate the same statistic.

## Implementation

The 20 metrics, identified in the literature review (Table 1) and defined in Section “Metric definitions and Algorithms”, have been implemented in a tool named EvaluateSegmentation and provided as an open source project. This section is organized as follows: In Section “Architecture”, we provide an overview of the general architecture of the project. Section “Compatibility” provides information about the compatibility of the tool with the image formats. Detail about the programming language, framework, and environment are provided in Section “Programming environment”. Some implementation details concerning the optimizations in the tool are presented in Section “Efficiency optimization”. Finally, Section “Usage” presents some cases of usage.

### Architecture

EvaluateSegmentation is an efficient command line tool that compares two 2D or 3D medical segmentations using the 20 evaluation metrics presented in Table 1. Being a pure command line tool without a GUI interface makes it suitable to be called using automation scripts when many segmentations are to be evaluated. The implementation has been generally designed to take advantage of the relations between the 20 implemented metrics represented in their definition in order to make use of the synergy between them to avoid repeating operations and hence to save execution time and memory. By default the evaluation result is displayed in a readable format on the System out, but it can be optionally saved as an XML file in a given path, e.g. to be parsed and processed by other tools.

### Compatibility

The proposed tool uses the ITK Library, in particular the input/output layer, to read medical images, which gives it two important properties:

- The tool is fully compatible with a wide spectrum of medical image formats, namely all formats supported by the ITK framework.
- The tool is invariant to changes in file formats, e.g. it is also compatible with formats that are changed, or even introduced after its implementation. That is because the job reading the images is done by the ITK library, which is permanently maintained to support new standards.

### Programming environment

EvaluateSegmentation is implemented in C++ using the CMake framework, which makes it operating system and compiler independent. CMake (www.cmake.org) is an open source platform that enables programs implemented in native languages like C++ to be operating system and compiler independent; it was originally created and funded by the National Library of Medicine (NLM) to provide a sufficient way for distributing the ITK application. The source of the project as well as builds for some operating systems are available under http://github.com/codalab/EvaluateSegmentation. To build the EvaluateSegmentation for any operating system, using any compiler, two resource components are required (i) the source code of the project and (ii) the ITK Library available as open source under http://www.itk.org.

### Efficiency optimization

Efficiency in speed as well as in memory usage is a critical point in metric calculation. Reasons for this are: (i) Very large 3D images, like whole body images, are quite common; such images could have more than 100 Mio voxels. (ii) Common image formats allow large data types for representing fuzzy voxel values, e.g. double, which makes the handling of such images memory critical. (iii) Metrics based on calculating the pairwise distances between all voxels become computationally inefficient with increasing volume size. (iv) State-of-the-art techniques based on the distance transform are sensitive to increasing image grid size in terms of speed as well as memory used.

EvaluateSegmentation doesn’t use distance transform techniques for calculations because of their memory sensitivity to grid size. Instead, it uses optimization techniques that make it very efficient in terms of speed and memory: To overcome the memory problem of large images with large data types, in a first step, EvaluateSegmentation uses a streaming technique, supported by ITK, to load images and save them in another representation that supports values in 255 fuzzy levels using the char data type; thereby overcoming the memory problem with large data types. In a next step, EvaluateSegmentation uses indexing techniques to model the images in a way that (i) makes use of excluding the background voxels, which makes the tool less sensitive to increasing the grid size, (ii) provides an image representation that is optimal for an efficient access to the image, and uses optimization techniques for calculating nearest neighbor operations.

The Hausdorff distance (*HD*) and the average Hausdorff distance (*AVD*) are based on calculating the distances between all pairs of voxels. This makes them computationally very intensive, especially with large images.

For the *HD*, EvaluateSegmentation uses the randomization and the early breaking optimizations proposed in [39] to achieve efficient, almost linear, calculation. These optimizations avoid scanning all voxel pairs by identifying and skipping unnecessary rounds.

**Fig. 1:**
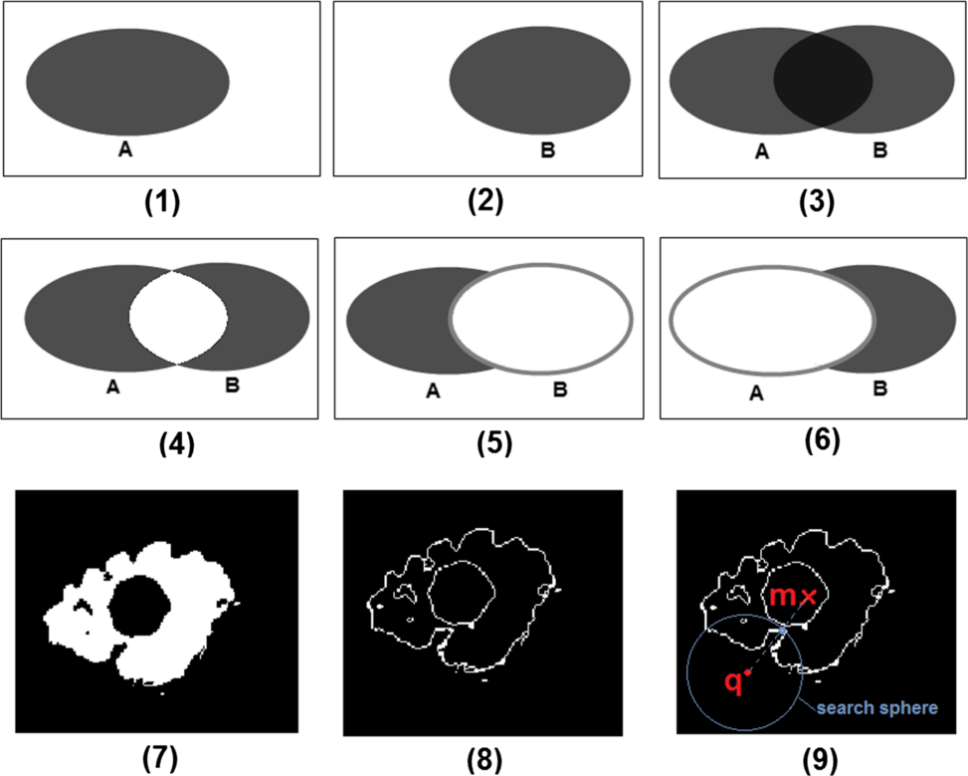
Illustration of the optimizations used in calculating the average distance(*AVD*). In **1** and **2**, the images A and B, defined on the same grid, are to be compared using the *AVD*. In **3**, the intersection of the images is identified. In **4**, the pairwise distance between point in the intersection is zero, therefore these distances are excluded from the calculation. In **5**, to find the minimum distance from a point in A to the the image B, only the boundary voxels of B are considered. In **6**, likewise to find the minimum distance from a point in B to the A, only the boundary voxels of A considered. In **7** and **8**, the boundary voxels of a real segmentation of the edema of a brain tumor. In **9**, to reduce the search space when searching the nearest neighbor, a search sphere with radius *r* is found by moving from the query *q* toward the mean *m* and considering the first point crossed on the boundary

Unfortunately, these two optimizations cannot be applied for the *AVD* because *AVD* attempts to calculate all the *HD* distances and finally considers their average. Therefore, to efficiently calculate the *AVD*, we use a modified version of the nearest neighbor (NN) algorithm proposed by Zhao et al. [42] in which a 3D cell grid is built on the point cloud and for each query point, a search subspace (a subset of the cell grids that contains the nearest neighbor) is found to limit the search and reduce the number of distance calculations needed. We add three modifications to this algorithm that achieve an optimal efficiency in finding the exact NN. These modifications make use of the nature of segmentations, namely that they are mostly dense point clouds. In the first modification, when calculating the pairwise distances from segment *A* to *B*, as illustrated in Fig. 1 (1) to (4), we remove the intersection *A*∩*B* from consideration because here all the distances are zero, that is we calculate only *A*∖*B* to *B*. For the second modification, instead of considering all points of *B*, we consider only the points on the surface of segment *B* as illustrated in Fig. 1 (5) and (6). This is justified by the fact that when moving in a line from a point in segment *A* (but not in the intersection) to the segment *B*, the first point crossed in *B* is on the surface and this is the shortest distance, which means all points inside the segments are not relevant. Figure 1 (7) and (8) illustrate a real segmentation of the edema of a brain tumor and the boundary voxels of the segmented edema. The third modification is to find the radius *r* that defines a convenient search subspace for a given query point *q*∈*A*, as illustrated in Fig. 1 (9). We find *r* by moving from *q* to the mean of *B* (*m*) and if a point *p*∈*B* is crossed, we define *r* as the distance between *q* and *p*, i.e. the search subspace consists of all cell grids contained in or crossed by the sphere centered on *q* with radius *r*. If no point *p* is found (which is unlikely to happen with segmentations), an exhaustive search is performed.

### Usage

EvaluateSegmentation is a command line tool. The command line has a mandatory part specifying the two images being compared and an optional path with arguments used to control the metric calculation. The command line has the following syntax:

EvaluateSegmentation groundtruthpath segmentationpath [-thd threshold] [-use DICE,JAC,HD,....] [-xml xmlpath]

By default, unless other options are given, a fuzzy comparison is performed, otherwise if a threshold, option -thd, is given, binary representations of the images are compared by cutting them at the given threshold. All metrics are considered unless the option -use is given, which specifies the metrics to be calculated. In this case, the symbols of metrics of interest, according to Table 1, should be listed after the option, separated with commas. Some metrics use parameters like the quantile value of the Hausdorff distance; these parameters can be optionally written following the metric symbol after an @, e.g. -use HD@0.9 instructs the tool to calculate the Hausdorff distance at 0.9 quantile. More options are described by typing EvaluateSegmentation at the command line.

## Results and discussion

This section is organized as follows: In Section “Testing the efficiency”, we present experiments that test the efficiency of the proposed evaluation tool. In Section “Results and discussion”, we present a discussion of the metrics implemented in this tool, by analyzing their properties and relating them to properties of the segmentations as well as to the requirements on the segmentation algorithms. Based on this analysis, we conclude guidelines for selecting the most suitable metric for given image data and segmentation task.

### Testing the efficiency

We present the experiments that validate the efficiency of the proposed evaluation tool (EvaluateSegmentation) with two different sets of real MR and CT volume segmentations. In the first two experiments (Sections Efficiency test with brain tumor segmentation to Efficiency test with whole body volumes), the proposed tool was tested against the implementation of the evaluation algorithms of the ITK library version 4.4.1, assumed to represent the state-of-the-art. These ITK algorithms are based on the distance transform technique, described in [51] and [52]. Only two metrics were considered, namely the Hausdorff distance (*HD*) and average distance (*AVD*) because they are the most time and memory-consuming metrics. This was controlled by using the command line options to limit the calculation to these metrics. In the third experiment (Section “Efficiency of calculating 20 metrics together”), we test the efficiency of the proposed tool when performing all of the implemented metrics (20 metrics) to show the benefit of using the synergy, i.e. building on the group of basic values. All experiments were executed on a machine with Intel Core (i5) CPU, 8 GB RAM and Windows 7 OS. Note that all execution times include the time for reading the images and calculating the metrics.

#### Efficiency test with brain tumor segmentation

**Fig. 2:**
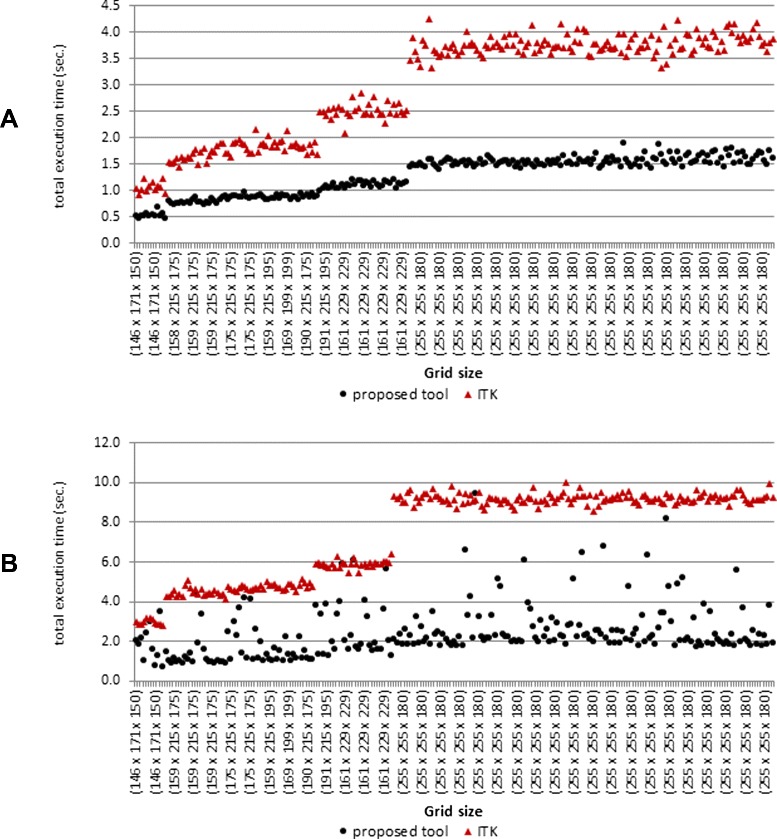
Testing the proposed tool against the ITK implementation using brain tumor segmentation. Comparison between the performance of the proposed evaluation tool and the ITK Library implementation in validating 240 brain tumor segmentations against the corresponding ground truth using the *HD* in (**a**) and the *AVD* in (**b**). The grid size (*w* *i* *d* *t* *h*×*h* *e* *i* *g* *h* *t*×*d* *e* *p* *t* *h*) is on the horizontal axis and the run time in seconds is on the vertical axis. The data points are sorted according to the total number of voxels, i.e. *w* *h* *d*

In this experiment, the proposed evaluation tool (EvaluateSegmentation) was tested with brain tumor segmentations (MR 3D images). We used a test set of 300 automatic brain tumor segmentations from the BRATS2012 challenge^4^. The test set consists of 240 images and 60 ground truth segmentations made by human experts. These images were produced by segmentation algorithms proposed by four participants of the BRATS challenge. The images vary widely in size and span the range from 125×125×125 to 250×250×250 voxels as grid size. Each of these images was compared with the corresponding ground truth segmentation using the Hausdorff distance *HD* in one run and the average distance *AVD* in another run. Figure 2() shows that the proposed tool outperforms the ITK implementation in computing the *HD* by a factor of 2.4 and takes an average runtime of 1.3 s. Figure 2() shows that the proposed tool outperforms the ITK implementation in computing the *AVD* by a factor of 3.0 and takes an average of 2.5 s. Furthermore, the experiment shows that while the efficiency of the proposed evaluation tool depends mainly on the set size (size of the segments), the efficiency of the ITK implementation is also strongly dependent on the grid size of the volumes, which makes it sensitive to increasing the grid size, which is more clear in the experiment in Section “Efficiency test with whole body volumes”.

#### Efficiency test with whole body volumes

In this experiment we test the runtime behavior of the proposed evaluation tool when the grid size of the 3D image is increased. For this, we tested it with very large 3D MR and CT image segmentations from the VISCERAL project [53]. The set consists of 840 MRI and CT 3D image segmentations. These were produced by segmentation algorithms proposed by five participants of the VISCERAL Anatomy 1 Benchmark. The images span the range from 387×21×1503 to 511×511×899 voxels as grid size. Each of these images was validated against the corresponding ground truth segmentation using the *AVD*. In a first run, the tool EvaluateSegmentation was executed and in a second run, the algorithm of the ITK Library. The proposed tool ran through successfully with all images, with execution times varying from 2.1 s for the smallest image to 79.2 s for the largest, giving an average runtime of 39.8 s over all images. The ITK algorithm broke down with a memory allocation error with all images over 387×25×1509, which means that only 17 *%* of the images have been successfully compared by the ITK algorithm. The failing of the ITK implementation with images with large grid size can be explained by the fact that the distance transform based algorithms are sensitive to increasing grid size because all the background voxels should be labeled. On the contrary, the algorithms used in the proposed evaluation tool are not sensitive to grid size increase because the background is not involved in the computation at all.

#### Efficiency of calculating 20 metrics together

In this experiment, we test the efficiency of the evaluation tool when calculating all implemented metrics together in one run. To this end, we used the same image set as in SubSection “Efficiency test with whole body volumes”. The proposed evaluation tool was executed to compare each of the segmentations with the corresponding ground truth segmentation, this time using all 20 implemented metrics. In each comparison, the total execution time was measured, which includes the time needed to read and preprocess the image as well as calculate all the metrics. The proposed tool takes a minimum runtime of 2.1 s, a maximum of 94.5 s, and an average runtime of 43.2 s to compare medical volumes using all implemented metrics. Note that this execution time is only slightly more than the time needed to calculate the *AVD* alone. This is possible due to using the synergy between metrics, e.g. building on basic values to avoid unnecessary calculations and repeated read operations.

### Metric selection

After we have defined a metric pool of 20 metrics, and provided an efficient implementation for calculating these metrics, we provide in this section guidelines for selecting a subset of these metrics depending on the segmentation being evaluated and the segmentation goal. Metrics differ in their properties and thus in their suitability for different tasks and different data. Selecting a suitable metric is not a trivial task.

We will define guidelines for selecting evaluation metrics in the following steps: (i) We provide metric analysis in Setion 1, based on examining the correlation among the metrics under different situations, providing empirical examples, and considering notes and results in the literature. As results of this analysis, we provide in Section “Metric properties” definitions of metric properties and we match them to the metrics in Table 1. (ii) In a second step, we define in Section “Segmentation properties” properties that the segmentations, being evaluated, can have. In Section “Requirements on the segmentation algorithms” we define the requirements that can be put on the segmentation algorithm. (ii) Finally, based on these properties and requirements, we provide in Section “Guidelines for selecting evaluation metrics” guidelines for metric selection in the form of a protocol that provides recommendation or discouragement for particular combinations of metric properties, data properties, and requirements.

#### Metric analysis

In this section, we analyze the metrics in Table 1 to infer their properties, i.e. their strength, weakness, bias, and sensitivities in evaluating medical segmentation. For this, we use two strategies, the first is examining the correlation between rankings of segmentations produced by different metrics in different situations. The second method is analyzing the metric values for particular empirical examples, where the segmentations have particular properties.

##### Correlation among metrics

**Fig. 3:**
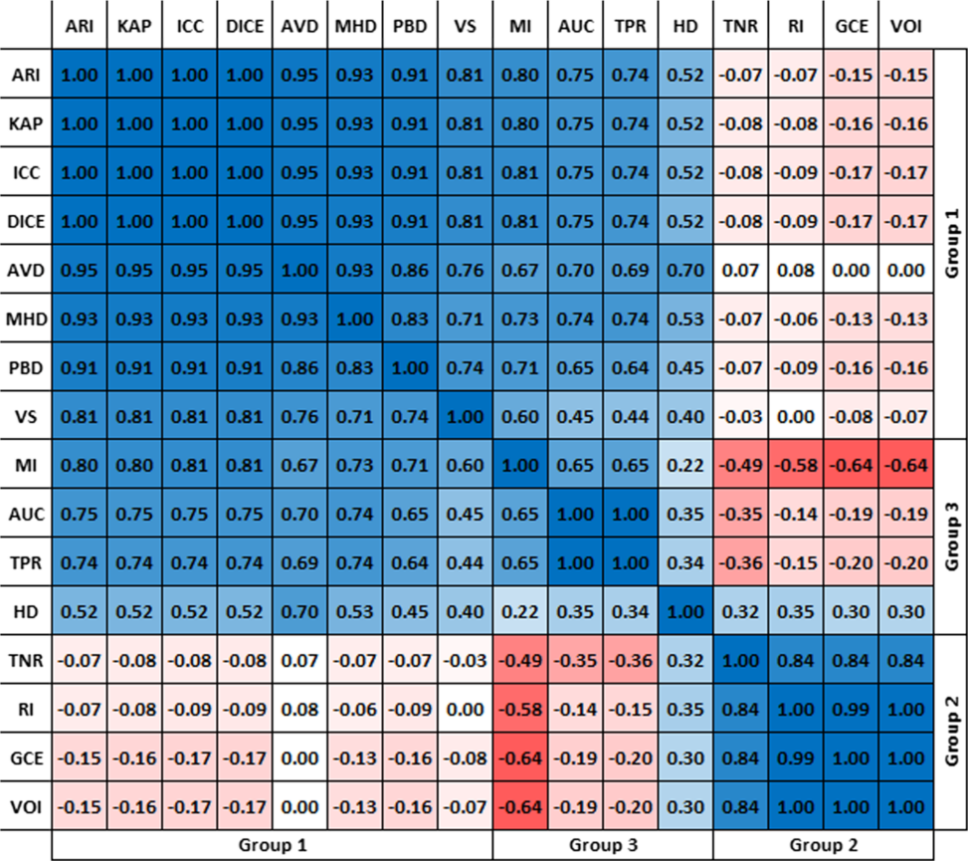
The correlation between the rankings produced by 16 different metrics. The pair-wise Pearson’s correlation coefficients between the rankings of 4833 medical volume segmentations produced by 16 metrics. The color intensity of each cell represents the strength of the correlation, where blue denotes direct correlation and red denotes inverse correlation

In this section, we examine the correlation between rankings of segmentations produced by different metrics without putting any constraints on the segmentations being ranked. Figure 3 shows the result of a correlation analysis between the rankings produced by 16 of the metrics presented in Table 1 when applied to a data set of 4833 automatic MRI and CT segmentations. In this data set, all medical volumes provided by all the participants in the VISCERAL project [53] Anatomy 1 and Anatomy 2 Benchmarks were included. Each medical image is a segmentation of only one of 20 anatomical structures varying from organs like lung, liver, and kidney to bone structures like vertebra, glands like thyroid, and arteries like aorta. More details on these structures are available in [54]. Note that the Jaccard (*JAC*) and F-Measure (*FMS*) were excluded because they provide the same ranking as the Dice coefficient (*DICE*), a fact that follows from the equivalence relations described in Section “Calculation of overlap based metrics”. Also *FPR* and *FNR* were excluded because of their relations to *TNR* and *TPR* respectively, as given in Eqs. 12 and 13. In a first step, volume segmentations were ranked using each of the metrics to get 16 rankings in total. Then, the pairwise Pearson’s correlation coefficients were calculated. Note that analyzing the correlation between rankings instead of metric values solves the problem that some of the metrics are similarities and some others are distances and avoids the necessity to convert distances to similarities as well as to normalize metrics to a common range. Each cell in Fig. 3 represents the Pearson’s correlation coefficients between the rankings produced by the corresponding metrics. The color intensity of the cells represent the strength of the correlation.

**Fig. 4:**
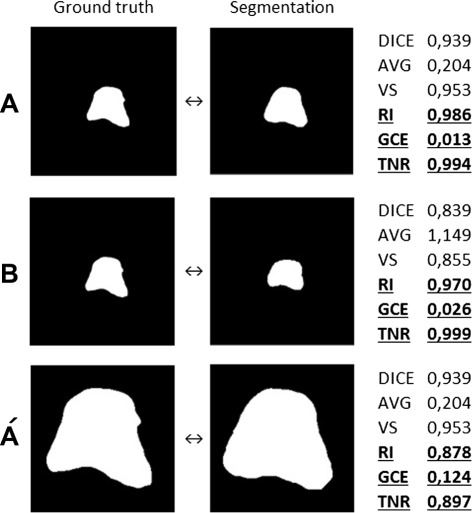
The effect of decreasing the true negatives (background) on the ranking. Each of the segmentations in *A* and *B* is compared with the same ground truth. All metrics assess that the segmentation in *A* is more similar to the ground truth than in *B*. In $\acute {A}$, the segmentation and ground truth are the same as in *A*, but after reducing the true negatives by selecting a smaller bounding cube. The metrics *RI*, *GCE*, and *TNR* change their rankings as a result of reducing the true negatives. Note that some of the metrics are similarities and others are distances

Metrics in Fig. 3 can be divided into three groups based on the correlation between the rankings produced by them, one group is at the top left (Group 1) including *ARI*, *KAP*, *ICC*, *DICE*, *AVD*, *MHD*, *PBD*, and *VS* and another group is at the right bottom (Group 2) including *TNR*, *RI*, *GCE*, and *VOI*. The metrics in each of these groups strongly correlate with each other, but have no correlation with metrics in the other group. The remaining metrics (Group 3) including *MI*, *AUC*, *TPR*, and *HD* have medium correlation between each other and the other groups. A deeper consideration in the metric definitions shows that Group 1 and Group 2 classify the metrics according to whether they consider or do not consider the true negatives (background voxels) in their definitions. While all metrics in Group 2 include the true negatives in their definitions, none of the metrics in Group 1 does this. Note that the adjusted Rand index and the kappa measures principally include the true negatives in their definitions, but both of them perform chance adjustment, which eliminates the impact of the true negatives, i.e. avoids that the influence of the background dominates the result [55]. Also note that the average distance (*AVD*) and the Mahalanobis distance (*MHD*) in Group 1 do not consider the true negatives, since they are based on the distances between the foreground voxels (non-zero voxels). Considering the true negatives in the evaluation has a large impact on the result, since the background (normally the largest part of the segmentation) contributes to the agreement. Figure 4 illustrates, by means of a real example, how metrics based on the true negatives change the resulting rankings when the true negatives are reduced by selecting a smaller bounding cube [10]. Such metrics are biased against the ratio between the total number of foreground voxels and the number of the background voxels, which is denoted as the class imbalance. This leads to segmentations with large segments being penalized and those with small ones being rewarded, a case that is common in medical image segmentation e.g. when the quality of two segmentations is to be compared, where one of them is larger, and the other one is smaller than the ground truth segmentation. Vinh et al. [7] stated that such metrics need chance adjustment, since they do not meet the constant baseline property.

##### Effects of overlap on the correlation

Obviously, the correlation between rankings produced by overlap based metrics and rankings produced by distance based metrics cannot hold in all cases. For example, consider the case where the overlap between segments is zero, here all overlap based metrics provide zero values regardless of the positions of the segments. On the contrary, distance based metrics still provide values dependent on the spatial distance between the segments. This motivated us to examine how the correlation described in Section “Correlation among metrics” behaves when only segmentations with overlap values in particular ranges are considered.

**Fig. 5:**
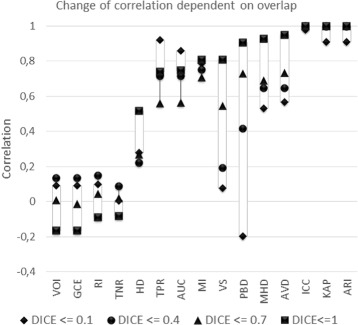
The effect of overlap on the correlation between rankings produced by different metrics. The positions and heights of the bars show how metrics correlate with *DICE* and how this correlation depends on the overlap between the compared segmentations. Four different overlap ranges are considered

Figure 5 shows the Pearsons’s correlation between the *DICE* and each of the other metrics when the measured *DICE* is in a particular range. One important observation is that the correlation between *DICE* and the distance based metrics (*AVD*, *HD*, and *MHD*) decreases with decreasing overlap, i.e. with increasing false positives and false negatives. This is intuitive because overlap based metrics, in contrast to distance based metrics, don’t consider the positions of voxels that are not in the overlap region (false positives and false negatives), which means that they provide the same value independent of the distance between the voxels. It follows that increasing the false positives and/or false negatives (decreasing overlap) means increasing the probability of divergent correlation.

Another observation is the strongly divergent correlation between volumetric similarity (*VS*) and *DICE*. This divergence is intuitive since the *VS* only compares the volume of the segment(s) in the automatic segmentation with the volume in the ground truth, which implicitly assumes that the segments are optimally aligned. Obviously, this assumption only makes sense when the overlap is high. Actually, the *VS* can have its maximum value (one) even when the overlap is zero. However, the smaller the overlap, the higher is the probability that two segments that are similar in volume are not aligned, which explains the strong divergence in correlation when the overlap is low.

Finally, the highest divergence in the correlation is observed with the probabilistic distance (*PBD*). This is caused by the fact that *PBD*, in contrast to *DICE*, over-penalizes false positives and false negatives. This can be explained by means of the definition of the *PBD* in Eq. 42: differences in the voxel values in the compared segmentations have a double impact on the result because they increase the numerator and decrease the denominator at the same time, causing the distance to increase rapidly. Actually, the *PBD* even reaches infinity when the overlap reaches zero. *PBD* behaves the opposite of the *VS* regarding the sensitivity to the alignment, i.e. it strongly penalizes alignment errors (we mean with alignment errors that the segmented volume is correct, but the overlap is low). This makes *PBD* suitable for tasks where the alignment is of more interest than the volume and the contour.

##### Segment size:

There is an inverse relation between segment size (relative to the grid size) and the expectation value of the alignment error, which directly follows from the degree of freedom for the segment location being higher when the segment is small. Furthermore, there is a direct relation between the expectation of alignment error and overlap between the segment in the ground truth and that in the segmentation under test. For small segments, the expectation value of the alignment error can be comparable in magnitude with the segment size, which results in the probability of small (or zero) overlap being high. In such a case, all metrics based on the four overlap cardinalities (TP, TN, FP, FN), e.g. the overlap based metrics, are not suitable, since they would provide the same value regardless of how far the segments are from each other, once the overlap is zero. Obviously, metrics based on the volume, e.g. the volumetric similarity have also the same drawback. Distance based metrics are the better choice when segments are small. We define small segments to be when the smallest dimension of the segment, i.e. *min*(*length*,*width*,*height*), is significantly less than the corresponding dimension of the grid on which the image is defined (e.g. less than 5 % of the corresponding grid dimension). Note that at least one dimension should be small. This means that also segments that are small in only one dimension (planar shape) or small in two dimensions (linear shape) can cause the same effect (i.e. the expectation value of the alignment error is comparable with smallest dimension). To illustrate this effect, consider comparing two lines using *DICE*. Assume that the lines have almost exact match, but the overlap is zero. Here, the *DICE* provides the same value (zero) for these two lines and for another two lines that are far from each other. The same holds for two planes or two points.

##### Boundary errors

Anatomy structures that are segmented can be of different grades of complexity in terms of boundary delimitation. They can vary from simple and smooth shapes, like a kidney, to irregular shapes, like tumors, but also branched and complex like the vessels of the eye retina. It depends on the goal of the segmentation, whether the exact delimitation of the boundary is important or not. For example, the boundary can be of importance when the goal is monitoring the progress of a tumor. In other cases, the goal is to estimate the location and the size or general shape of an anatomical structure, e.g. a lesion. Here the alignment and the extent are rather more important then the boundary. Another requirement could be maximizing the recall at the cost of the boundary delimitation, i.e. to ensure that the segmented regions contain (include) all of the true segment, e.g. when the goal is to remove a tumor. In this section, we analyze the metrics in terms of their capabilities of (i) penalizing boundary errors, (ii) rewarding recall, and (iii) discovering the general shape, thereby ignoring small details.

**Fig. 6:**
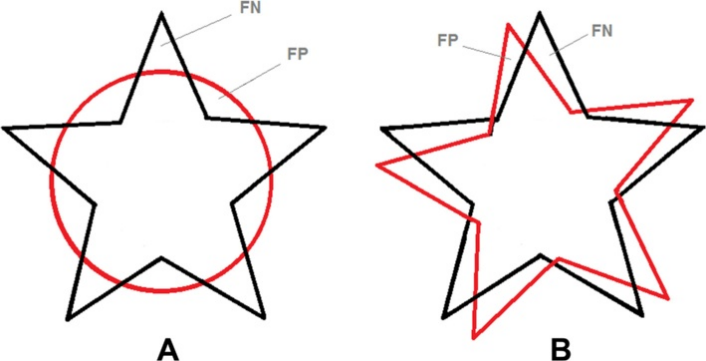
Metrics that fail to discover boundary errors. In **a**, the star is compared with a circle and in **b** the same star is compared with another star of the same dimensions, rotated so that the resulting overlap errors (FP and FN) are equal in magnitude in both cases. All metrics that are based on FP and FN (overlap-based metrics) are not able to discover that the two shapes in (**b**) are more similar to each other than those in (**a**). On the contrary, all spatial distance based metrics discover the similarity and give (**b**) a higher score than (**a**). However, the metric most invariant to boundary error is the volumetric similarity, since it gives a perfect match in both cases

Figure 6 illustrates the fact that metrics differently consider boundary delimitation. In (a) a star is compared with a circle and in (b), the same star is compared with another star that has the same shape and dimensions, but slightly rotated so that the resulting overlap errors FP and FN (obviously also the TP and TN) are the same as in (a). It follows that all metrics, defined based on the overlap error cardinalities, provide the same similarity between the two shapes in each case, which has been also confirmed empirically. This means that they do not discover that the shapes in (b) are more similar than those in (a), which also implies that such metrics are not recommended when segmentation algorithms are expected to provide accurate boundaries. However, the spatial based distance metrics, in particular the *HD* and the *AVD*, discover these boundary errors and provide higher similarity values for case (b). This makes these two metrics more suitable for cases where the boundary delimitation is of interest. Actually, as already mentioned in Section “Effects of overlap on the correlation”, this suitability follows from the fact that spatial based metrics consider the positions of the FP and FN in contrast to the overlap based metrics where FP voxels as well as FN voxels count the same regardless of their distances from the true positions. The volumetric similarity (*VS*) is also not recommended to discover boundary errors. Note that in (a) and (b), the *VS* provides a perfect match, given |*FP*|=|*FN*| regardless of the boundary. *VS* is recommended for cases where the segmented volume is in the focus of interest regardless of the boundary and the alignment.

##### Rewarding recall

**Fig. 7:**
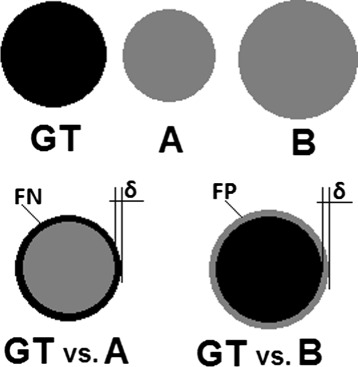
Boundary errors: rewarding/penalizing recall. Illustration in 2D of boundary errors that decrease/increase recall. The ground truth image GT is compared with the image A that is smaller than GT and with another image B that is larger than GT. Although the boundary error in both cases is equal (*δ*), the magnitude of the resulting false negative (FN) with A is smaller than the resulting false positive (FP) with B. This causes that metrics, considering the absolute magnitudes of FN and FP, penalize high racall

Segmentation errors can be due to missing regions (parts in the ground truth that are missing in the automatic segmentation) or added regions (parts in the automatic segmentation without corresponding parts in the ground truth). Depending on the application, sometimes missing regions harm more than added regions, which means that algorithms are preferred that aim to maximize recall on cost of precision, i.e. avoid missing regions, even on cost of having added regions. In this case, metrics that reward recall could be a good choice. Figure 7 illustrates in 2D how metrics differ in evaluating segmentations in terms of missing and added regions. In one case, the ground truth segment GT is compared with a smaller segment A and in another case GT is compared with a larger segment B. The distance between the boundary of the ground truth and the boundary of the segment *δ* is equal in both cases. However, the volume differences (FN and FP) are not equal, which causes metrics based on the four cardinalities (TP, TN, FP, FN) differently to evaluate the two cases. The metrics *MI* (mutual information) and *TPR* (recall) reward recall and hence evaluate B as better than A. This is because *MI* measures how much information the segmentation have in common, which obviously increases with recall.

##### Segmentation density

The density of segments in automatic segmentations can vary depending on the strategies used by the segmentation algorithms. While some algorithms produce solid segments, others produce segments with low density, e.g. due to a huge number of uniformly distributed tiny holes. It depends on the goal of the segmentation, whether the density of a segment is of importance or not. In some cases, the density has a meaning e.g. when it should measure the progress of a disease, and in other cases it is meaningless, e.g. when anatomical structures are to be localized, e.g. organs.

**Fig. 8:**
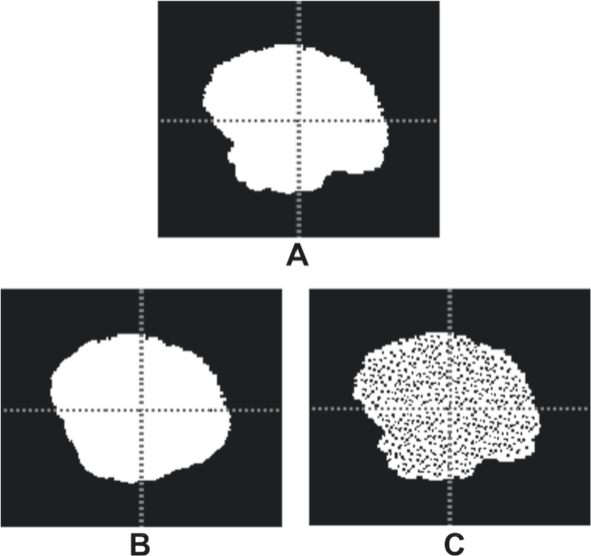
The effect of segment density. Two segmentations **b** and **c** are compared with the corresponding ground truth (**a**). **b** has a solid structure while **c** has a lower density due to large number of tiny holes uniformly distributed inside it. Although **c** has a a higher accuracy of the boundary than **b**, all metrics, excepts *MHD* and *HD*, give **b** a higher score than (**c**)

However, sometimes the density of the segments is not intended by the segmentation algorithm, but rather a side effect of the strategy used for the segmentation. There are cases where algorithms work very will in identifying the boundary of the structure being segmented, but produce segments with low density. Figure 8 shows a real example of brain tumor segmentation from the BRATS 2012 challenge, where a segmentation algorithm provides a solid segment (b) with low accuracy in identifying the boundary, and another algorithm (c) produces a segment with a boundary of higher accuracy, but the density is low due to numerous tiny holes. When comparing each of these cases with the corresponding ground truth (a), all the metrics, except the Mahalanobis distance (*MHD*) and the Hausdorff distance (*HD*), measure a higher similarity (or smaller distance) in (b) than in (c). The explanation is obvious, since all tiny holes are calculated as false negatives, which has impact on all metrics defined based on the four cardinalities (TP, TN, FP, FN). On the other hand, since the *MHD* estimates the general shape of the segment, thereby ignoring small details, it is not sensitive to segment density. Also the *HD* is not sensitive, since it is a maxi min operation, which means that errors caused by the tiny holes are ignored, when there exist larger errors. Given that the task is to identify the tumor core using a crisp segmentation, i.e. assigning each voxel either as tumor core or background, the question is whether it is justified to penalize the low density of the segment. However, in cases where the segment density is to be ignored, metrics with such sensitivity should be avoided.

##### General shape and alignment

The Mahalanobis distance *MHD* (Eqs 52 to 54) measures the distance between two segmentations by comparing estimates of them, in particular it considers the two ellipsoids that best represent the segmentations [43]. This way of comparison ignores the boundary details and considers only the general shape and the alignment of the segments. The could be a good choice when the goal of the exact shape of the segment is not a requirement.

#### Metric properties

Based on the results of the discussion so far, we summarize the properties of the metrics that are relevant for segmentation. In particular, we define these properties and assign them to the metrics listed in Table 1.

- *Outlier sensitivity:* Sometimes automatic segmentations have outliers in form of few pixels outside the segment. The underlying property describes metrics that strongly penalize such outliers.
- *True negatives consideration:* In a two class segmentation, the voxels are assigned either to the single segment or to the background. The voxels that are assigned as background by both the automatic segmentation and the ground truth are called the true negatives. The underlying property describes metrics that calculate the true negatives as a part of the agreement between the automatic segmentation and the ground truth.
- *Chance adjustment:* The agreement between two segmentations could be caused by chance. The score of a segmentation performed randomly, which is called the baseline, should ideally be zero. The underlying property describes metrics that have in their definition an adjustment to minimize the baseline value.
- *Sensitivity to point positions:* Some metrics, e.g. overlap-based metrics, do not consider the position of false positive voxels, i.e. they provide the same result wherever these voxels are. The underlying property describes metrics that do consider the position of the false positive, i.e. their values differ depending on where these voxels are.
- *Ignoring alignment errors:* alignment errors are when the segment in the automatic segmentation has similar shape and similar volume as the corresponding segment in ground truth, but it is not correctly aligned, e.g. translated or rotated. Some metrics are invariant to alignment error, i.e. they cannot discover them, like the volumetric similarity.
- *Recall rewarding:* Describes metrics that are not sensitive to errors increasing recall, in particular they penalize boundary errors that decrease the segmented volume more than errors that enlarge the segmented volume.
- *General shape and alignment:* Describes metrics that ignore small details and judge only the general shape and alignment of the segmented region.
- *Overlap-based:* This property describes metrics that are based on four types of overlap (TP, TN, FP, FN) between the automatic segmentation and the ground truth.
- *Distance-based:* This property describes metric that are defined as functions of the Euclidean distances between the voxels of the segment in the automatic segmentation and the voxels of the segment in the ground truth.
- *Information theoretical-based:* Describes metrics based on information theoretical factors like the entropy.
- *Probabilistic-based:* Describes metrics defined as functions of statistics calculated from the voxels in the overlap regions of the segmentations.
- *Pair-counting-based:* Considering that the segmentation is a partitioning of an image, pair-counting-based metrics consider grouping tuples representing all possible object pairs in four groups depending on where the objects of each pair are placed according to each of the partitions.
- *Volume-based:* Describes metrics that are defined based on the volume of the segmented region.

Now, depending on whether each of these properties holds or does not hold for a particular metric, we present the property assignments in Table 4, in which a check marked cell denotes that the corresponding metric has the corresponding property. This assignment will be used later in Section “Guidelines for selecting evaluation metrics” to define a protocol for selecting evaluation metrics.

**Table 4:** Assignment of properties to metrics. Assignment between the properties defined in Section “Metric properties” and the metrics defined in Table 1

	Outlier	True	Chance	Sensitive	Ignoring	Recall	General	Overlap-	Distance-	Information	Probabilistic-	Pair-	Volume-
	sensitive	negatives	adjustment	to point	alignment	rewarding	shape &	based	based	theoretical	based	counting-	based
		consideration		positions	errors		alignment					based	
DICE								✓					
JAC								✓					
TPR						✓		✓					
TNR		✓						✓					
FPR								✓					
FNR								✓					
FMS								✓					
VS					✓								✓
GCE		✓											
RI		✓										✓	
ARI		✓	✓									✓	
MI		✓				✓				✓			
VOI		✓								✓			
ICC		✓	✓								✓		
PBD											✓		
KAP		✓	✓								✓		
AUC		✓									✓		
HD	✓			✓					✓				
AVD				✓					✓				
MHD				✓			✓		✓				

A particular metric has a particular property iff the corresponding cell is check marked

#### Segmentation properties

Metric selection should consider, among others, the properties of the segmentations being evaluated. In this section, we define some of the properties that segmentations can have, to which metrics can be sensitive. These properties will be used in combination with the metric properties to define a protocol for metric selection in Section “Guidelines for selecting evaluation metrics”.

- *Outliers:* In segmentation, outliers are relatively small wrongly segmented regions outside (normally far from) the segment. Metrics sensitive to outliers over-penalize them. When outliers do not harm, metrics with sensitivity to outliers, such as the *HD*, should be avoided.
- *Small segment:* When a segment size is significantly smaller than the background, so that it is comparable in magnitude with the expectation of the alignment error, then all metrics based on the four overlap cardinalities (TP, TN, FP, FN), e.g. the overlap based metrics, as well as volume based metrics (*VS*) are not suitable. Small segments are those with at least one dimension being significantly smaller than the corresponding dimension of the grid on which the image is defined (e.g. less than 5 % of the corresponding grid dimension). In this case, distance based metrics are recommended.
- *Complex boundary:* While some segments have nearly round shape or smooth boundaries, there are others that have a non-regular shaped complex boundary, which are denoted by this property. Metrics that are sensitive to point positions (e.g. *HD* and *AVD*) are more suitable to evaluate such segmentation than others. Volume based metrics are to be avoided in this case.
- *Low densities:* Some algorithms produce segmentations that have a good quality in terms of contour and alignment, but the segments are not solid, but rather have a lower density, e.g. because of numerous tiny holes. All metrics based on the four cardinalities are sensitive to segment density. They penalize low density and hence should be avoided in cases where the low density does not harm. In these cases, distance based metrics (*HD*, *AVD*, and *MHD*) are good choices.
- *Low segmentation quality:* This property describes segmentations that have in general a low quality, i.e. it can be assumed that the segments have in general low overlap with the corresponding segments in the ground truth segmentation. When the overlap is low, distance based metrics are more capable of differentiating between segmentation qualities than volume based metrics. The volumetric similarity *VS* should be avoided.

#### Requirements on the segmentation algorithms

Depending on the goal of the segmentation, there could be special requirements on the segmentation algorithms. Many different requirements could be defined, which can strongly differ from case to case. In the following are some of the requirements that could be put on the segmentation algorithms.

- *Contour is important:* Depending on the individual task, the contour can be of interest, that is the segmentation algorithms should provide segments with boundary delimitation as exact as possible. Metrics that are sensitive to point positions (e.g. *HD* and *AVD*) are more suitable to evaluate such segmentation than others. Volume based metrics are to be avoided in this case.
- *Alignment is important:* When the requirement is the location (general alignment) of the segment rather than the boundary delimitation. In this case, the volume based metrics are not a good choice.
- *Recall is important:* In some cases, it is an important requirement that the segmented region includes at least all the true segment, regardless of including parts of the false region. Obviously, the boundary delimitation in this case is of less interest, and the algorithms should rather maximize the recall. Metrics that reward recall are the mutual information *MI* and the true positive rate *TPR*.
- *Volume is important:* Sometimes the magnitude of the segmented region is of more importance than the boundary and the alignment. Here, algorithms should segment region to have a volume as near to that of the true segment as possible. The volumetric similarity *VS* is recommended.
- *Only general shape and alignment:* The exact boundary and high overlap are not always requirements. Depending on the goal, sometimes the general shape and the alignment (location) are sufficient, e.g. when the requirement is to identify lesions and give an estimation of the size. For this case, the Mahalanobis distance *MHD* is a good choice.

#### Guidelines for selecting evaluation metrics

As has been stated in Section “Background”, different metrics have sensitivities to different properties of the segmentations, and thus they can discover different types of error. Taha et al. [56] provide a formal method for choosing the most suitable metric, given a set of segmentations to be evaluated and a segmentation task.

Now, we provide guidelines for choosing a suitable metric based on the results so far. These guidelines are additionally summarized in Table 5 in form of matching between data properties, requirements, and metric properties: (i) When the objective is to evaluate the general alignment of the segments, especially when the segments are small (the overlap is likely small or zero), it is recommended to use distance based metrics rather than overlap based metrics. The volumetric similarity (*VS*) is not suitable in this case. (ii) Distance based metrics are recommended when the contour of the segmentation, i.e. the accuracy at the boundary, is of importance [6]. This follows from being the only category of metrics that takes into consideration the spatial position of false negatives and false positives. (iii) The Hausdorff distance is sensitive to outliers and thus not recommended to be used when outliers are likely. However, methods for handling the outliers, such as the quantile method [41], could solve the problem, otherwise the average distance (*AVG*) and the overlap based metrics as well as probabilistic based metrics are known to be stable against outliers. (iv) Probabilistic distance (*PBD*) and overlap based metrics are recommended when the alignment of the segments is of interest rather than the overall segmentation accuracy [2]. (v) Metrics considering the true negatives in their definitions have sensitivity to segment size. They reward segmentations with small segments and penalize those with large segments [10]. Therefore, they tend to generally penalize algorithms that aim to maximize recall and reward algorithms that aim to maximize precision. Such metrics should be avoided in general, especially when the objective is to reward recall (vi) When the segmentations have a high class imbalance, e.g. segmentations with small segments, it is recommended to use metrics with chance adjustment, e.g. the Kappa measure (*KAP*) and the adjusted rand index (*ARI*) [29, 55]. (vii) When the segments are not solid, but rather have low densities, then all metrics that are based on volume or on the four cardinalities (TP, TN, FP, FN), are not recommended. In such cases distance-based metrics, especially *MHD* and *HD*, are recommended. (viii) Volumetric similarity is not recommended when the quality of the segmentations being evaluated is low in general, because the segments are likely to have low overlap with their corresponding segments in the ground truth. In this case, overlap-based and distance-based metrics are recommended. (ix) When the segmented volume is of importance, volumetric similarity and overlap based metrics are recommended rather than distance based-metrics. (x) When more than one objective is to be considered, which are in conflict, then it is recommended to to combine more than one metric, so that each of the objective is considered by one of the metrics. Thereby, it is recommended to possibly avoid selecting metrics that are strongly correlated (Fig. 3).

**Table 5:** Summary of metric selection guidelines

	DICE	JAC	TPR	TNR	FPR	FNR	FMS	VS	GCE	RI	ARI	MI	VOI	ICC	PBD	KAP	AUC	HD	AVD	MHD
Outliers exist	✓	✓					✓	✓				✓	✓			✓	✓	X	✓	✓
Small segment	X	X	X	X	X	X	X			X	X	X	X			X	X	✓	✓	✓
Complex boundary								X										✓	✓	X
Low densities	X	X	X	X	X	X	X	X	X	X	X	X	X	X	X	X	X	✓	✓	✓
Low segmentation quality								X										✓	✓	✓
Contour is important								X										✓	✓	X
Alignment is important								X												
Recall is important			✓									✓								
Volume is important								✓												
General shape & alignment	X	X	X	X	X	X	X	X	X	X	X	X	X	X	X	X	X	X	X	✓

Each row corresponds to either a segmentation property or a requirement and each column corresponds to one of the metrics in Table 1. A checked cell ($\checkmark $) denotes that the metric is recommended for the corresponding property/requirement, a crossed cell (X) denotes that the metric is not recommended, and empty cells denote neutrality

## Conclusion

We propose an efficient evaluation tool for 3D medical image segmentations using 20 evaluation metrics. These metrics are selected based on a comprehensive literature review about validation of medical images segmentations. The aim of this tool is to provide a standard for evaluating medical image segmentation by providing a consistent set of metrics. The proposed evaluation tool is implemented in the open source project “EvaluateSegmentation” available for download from http://github.com/codalab/EvaluateSegmentation. The implementation of this tool uses efficient techniques which make it address the challenges in the evaluation of medical segmentations. The algorithms used to calculate the metrics were selected and optimized to achieve high efficiency in speed and memory required to meet the challenging requirements of evaluating images with large grid size, like the whole body scans.

Since metrics have different properties (biases, sensitivities), selecting suitable metrics is not a trivial task. This paper provides analysis of the 20 implemented metrics, in particular of their properties, and suitabilities to evaluate segmentations, given particular requirements and segmentations with particular properties. This analysis is concluded by providing guidelines for selecting a subset of the implemented metrics, given segmentation properties and requirements.

## Availability and requirements

- **Project name:** EvaluateSegmentation
- **Project home page:**http://github.com/codalab/EvaluateSegmentation
- **Operating system(s):** Platform independent
- **Programming language:** C++ / CMake
- **Other requirements:** ITK Library available under http://www.itk.org
- **License:** Apache License Version 2.0, January 2004
- **Any restrictions to use by non-academics:** none

## Endnotes

^1^ More about TREC_EVAL under http://trec.nist.gov/trec_eval/

^2^ National Library of Medicine Insight Segmentation and Registration Toolkit (ITK) www.itk.org

^3^*FMS*_*β*_ can be derived by setting $\alpha =\frac {1}{\beta ^{2}+1}$ in Rijsbergen’s effectiveness measure $E=1-\frac {1}{\alpha \frac {1}{PPV} + (1-\alpha) \frac {1}{TPR}}$.

^4^ MICCAI 2012 Challenge on Multimodal Brain Tumor Segmentation, http://www2.imm.dtu.dk/projects/BRATS2012

## Abbreviations

ARI: Adjusted rand index
AUC: Area under ROC curve
AVD: Average distance
BRATS: Brain tumor segmentation
CT: Computed tomography
DICE: Dice coefficient
FMS: F-Measure
FN: False negative
FNR: False negative rate
FP: False positive
FPR: False positive rate
GCE: Global consistency error
HD: Hausdorff distance
ICC: Interclass correlation
ITK: Insight Segmentation and Registration Toolkit
JAC: Jaccard index
KAP: Cohens Kappa measure
MHD: Mahalanobis distance
MI: Mutual information
MR/MRI: Magnetic resonance image
NLM: National Library of Medicine
PBD: Probabilistic distance
RI: Rand index
TN: True negative
TNR: True negative rate
TP: True positive
TPR: True positive rate
VOI: Variation of information
VS: Volumetric similarity
VISCERAL: visual concept extraction challenge in radiology
